# Potential role of compost mixed biochar with rhizobacteria in mitigating lead toxicity in spinach

**DOI:** 10.1038/s41598-020-69183-9

**Published:** 2020-07-22

**Authors:** Muhammad Zafar-ul-Hye, Muhammad Tahzeeb-ul-Hassan, Muhammad Abid, Shah Fahad, Martin Brtnicky, Tereza Dokulilova, Rahul Datta, Subhan Danish

**Affiliations:** 1 Department of Soil Science, Faculty of Agricultural Sciences and Technology, Bahauddin Zakariya University, Multan, 60800 Punjab Pakistan; 2 Department of Agronomy, The University of Haripur, Haripur, 22620 Pakistan; 3 College of Plant Sciences and Technology, Huazhong Agriculture University, Wuhan, China; 4 Department of Agrochemistry, Soil Science, Microbiology and Plant Nutrition, Faculty of AgriSciences, Mendel University in Brno, Zemedelska 1, 61300 Brno, Czech Republic; 5 Institute of Chemistry and Technology of Environmental Protection, Brno University of Technology, Faculty of Chemistry, Purkynova 118, 62100 Brno, Czech Republic

**Keywords:** Abiotic, Environmental impact, Bacterial secretion

## Abstract

Consumption of heavy metals, especially lead (Pb) contaminated food is a serious threat to human health. Higher Pb uptake by the plant affects the quality, growth and yield of crops. However, inoculation of plant growth-promoting rhizobacteria (PGPR) along with a mixture of organic amendments and biochar could be an effective way to overcome the problem of Pb toxicity. That’s why current pot experiment was conducted to investigate the effect of compost mixed biochar (CB) and ACC deaminase producing PGPR on growth and yield of spinach plants under artificially induced Pb toxicity. Six different treatments i.e., control, *Alcaligenes faecalis* (PGPR1), *Bacillus amyloliquefaciens* (PGPR2), compost + biochar (CB), PGPR1 + CB and PGPR2 + CB were applied under 250 mg Pb kg^-1^ soil. Results showed that inoculation of PGPRs (*Alcaligenes faecalis* and *Bacillus amyloliquefaciens*) alone and along with CB significantly enhanced root fresh (47%) and dry weight (31%), potassium concentration (11%) in the spinach plant. Whereas, CB + *Bacillus amyloliquefaciens* significantly decreased (43%) the concentration of Pb in the spinach root over control. In conclusion, CB + *Bacillus amyloliquefaciens* has the potential to mitigate the Pb induced toxicity in the spinach. The obtained result can be further used in the planning and execution of rhizobacteria and compost mixed biochar-based soil amendment.

## Introduction

Heavy metals are a group of metal or metalloids that are toxic to animals and human being at even lower concentrations. They tend to accumulate in a living organism i.e., plants and animals, so their uptake is ultimately a severe threat to human health. Heavy metal contaminated soil adversely affect the growth and development of plant and microorganisms^1,2^, which results in a reduction of crop productivity^3^.

Among various heavy metals, lead (Pb) has become a significant soil contaminant. Although Pb is a non-essential element, it gets absorbed by the crop plants and inhibits plant growth^4^. A large portion of Pb in the soil may come from fertilizers and automotive exhaust^5^. Furthermore, anthropogenic activities are also playing an imperative role in the buildup of Pb contamination in air, soil and water^6^. Plants uptake Pb from the soil solution by roots, and it gets accumulation in an insoluble form within the roots^7^. Higher Pb contamination in the soil causes low nitrogen assimilation in plants^8^, reduces the rate of seed germination and alterations in plant water relations^9^. Carotenoid, chlorophyll contents, carbon dioxide, assimilation rate and photosynthetic rate are also reduced in plants due to Pb exposure^4^. However, Pb transportation is usually limited from roots to other parts of the plant^10^. Casparian strip present in endodermis is the main barrier to lead transport across the endodermis into vascular tissue^11^.

So far, various strategies have been examined by many research groups to mitigate Pb toxicity in plants^1,12^. However, the reports suggested that the use of activated black carbon biochar is largely effective in reducing heavy metals induced stress in crops. The use of biochar to absorb organic contaminants and heavy metals in the soil is a promising and low-cost solution to heavy metal toxicity ^13–15^. Biochar is gaining the attention of the scientists^16^ as it can immobilize heavy metal and reduce their bioavailability to plant^17^. Activated carbon biochar is an appropriate organic amendment for the alleviation of heavy metals induced stress in plants due to its high absorption ability for metallic ions. It has been well documented that the microporous structure, ion exchange capacity, and active functional groups of biochar and play an imperative role in decreasing the mobility and bioavailability of heavy metals^18^. Furthermore, the use of compost as an organic amendment also enhances the productivity of crops. Application of compost facilitates rhizobacterial proliferation, improves soil aggregation, water holding capacity, and pH when applied in the soil^19^.

In addition to compost and biochar, augmentation of plant growth-promoting rhizobacteria (PGPR) also produce a wide variety of molecules, which improves plant growth and productivity^20–28^. These PGPRs increased the production of phytohormones or other molecules that protect plants from biotic and abiotic stress, increases mineral nutrition, modulating ethylene levels in plants and production of volatile organic compounds^20,29^. Furthermore, PGPRs also promotes beneficial symbioses and degrades the xenobiotic to protect the plants^29,30^.

Spinach (*Spinacia oleracea* L.) is an essential dietary vegetable. It plays a vital role in the supply of micronutrients and providing potassium, iron, folic acid, magnesium and manganese, and vitamins, i.e., K, C, B_2_, A^31^. Spinach has high antioxidant activity, mainly related to the presence of flavonoids, which is a major constituent of water-soluble polyphenols^32^. High omega-3 fatty acids, vitamins (E & B_6_), and dietary fiber found in spinach are essential for the improvement, regulation, and maintenance of the tissues in humans^33^. However, spinach is a very good accumulator of metals, especially Pb^34^.

That’s why current study was conducted with aim to examine the combined effects of ACC deaminase producing rhizobacteria and compost mixed biochar (CB) regarding immobilization of Pb in spinach cultivated in artificially induced Pb-contaminated soil. We hypothesized that combined use of ACC deaminase producing rhizobacteria and CB could be a more effective strategy over the sole application for the improvement in spinach growth under Pb stress.

## Results and discussion

### Soil pHs

**Figure 1:**
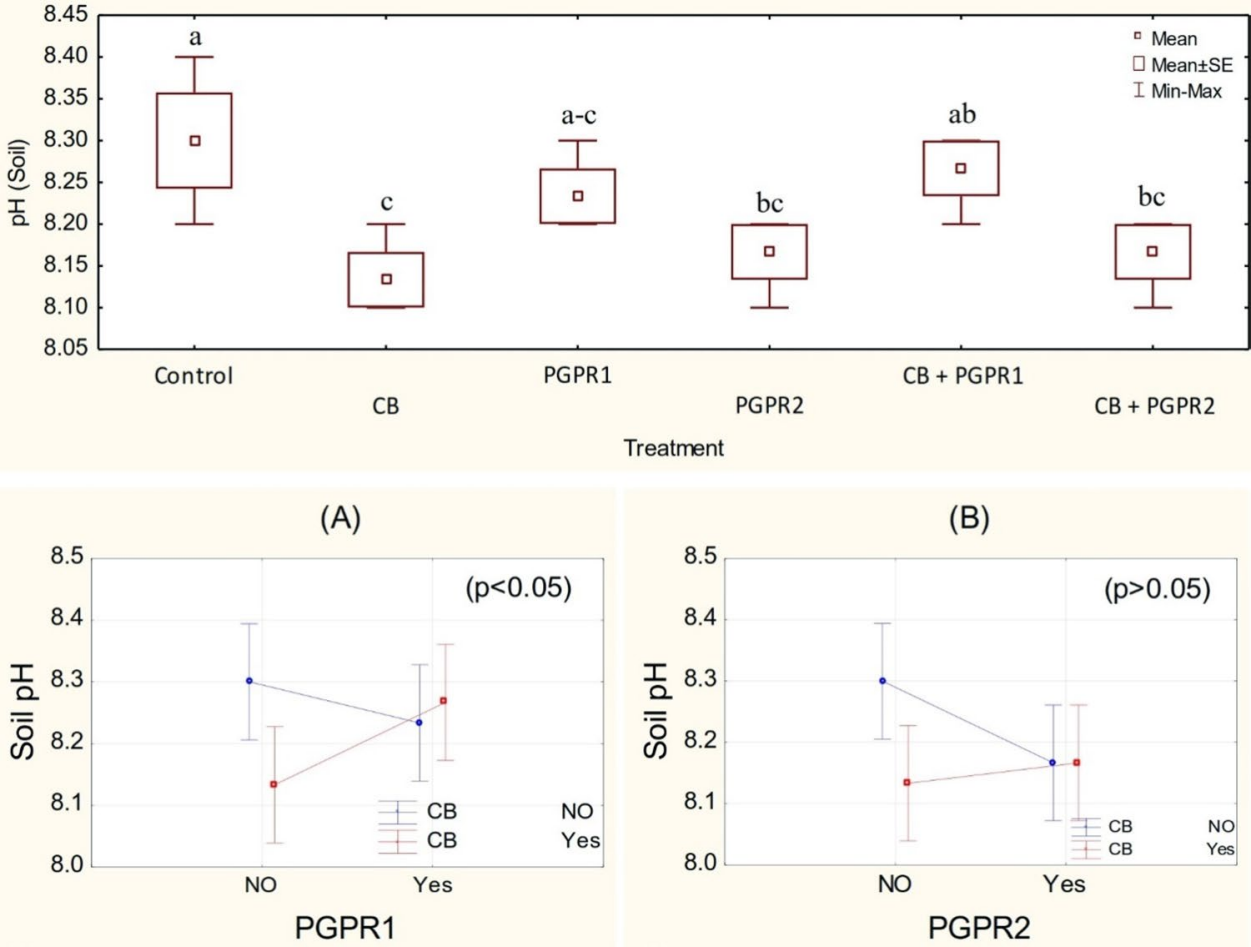
pH values of soil treated with CB, PGPR1, PGPR2 and their combination with CB. Means of three replicates having different small letters express significant differences at p ≤ 0.05 compared with Duncan’s test. Interaction graph of PGPR1 and CB **(A)**; PGPR2 and CB **(B)**, for soil pH*s*.

One-way analysis of variance between different treatments shows a significant (p ≤ 0.05) decrease in soil pH value as compared to control. It was observed that PGPR1 and CB have a significant (p ≤ 0.05) main effect on soil pH (Fig. 1), and a significant ordinal interaction was found between PGPR1 and CB (Fig. 1A). Inoculation of PGPR2 and CB do not have either their significant main effect or their interaction but the interaction was ordinal for soil pH (Fig. 1B). Application of CB remained significant regarding the decrease in soil pH as compared to the control. It was observed that PGPR2 also differed significantly from control for decreasing the soil pH. Treatment CB + PGPR2 remained statistically alike with PGPR2 and CB but differed significantly as compared to the control (Fig. 1). No significant change was noted over control in the soil pH where PGPR1 and CB + PGPR1 were applied. However, Maximum decrease of 2.0% in the soil pH was noted than control where CB was applied as an amendment. The reduction in the soil pH by applying CB occurred due to organic secretions of PGPR and low pH of compost as compared to biochar and soil (Table 1). The presence of microbes also secretes organic acids which play an imperative role in the solubilization of immobilized nutrients and decrease in pH of rhizosphere ^35^. Furthermore, decomposition of organic material i.e., compost also releases acidic compounds in the soil ^36^. Enrichment of humic acid in the rhizosphere by application of compost is another allied reason for a decrease in soil pH ^37^. In addition to above, the presence of water-soluble carbon compounds in compost are readily degradable by microbial acidic secretions which also contribute in decreasing the pH of soil ^38^.

**Table 1:** Pre-sowing analyses of soil and organic amendments.

Characteristics	Soil	Biochar	Compost	Characteristics	*B. amyloliquefaciens*	*A. faecalis*
Textural class	Loam	–	–	IAA with l-Tryptophan (µgml^-1^)	22.23	15.33
pH_***s***_	8.35	8.04	5.30			
EC_***e***_ (dS m^–1^)	1.05	3.49	–			
Organic matter (%)	0.31	–	–	IAA without l-Tryptophan (µgml^-1^)	5.63	2.21
Total nitrogen (%)	0.016	1.63	1.00			
Available phosphorus (mg kg^–1^)	3.42	0.40	0.53			
Extractable potassium (mg kg^–1^)	78	27	55	ACC deaminase α-ketobutyratenmol g^-1^protein h^-1^	232	484
Extractable lead (mg kg^–1^)	0.51	2.09	1.15			
Volatile matter (%)	–	14.4	–	Exopolysaccharide	+	+
Ash content (%)	–	16.8	–			
Fixed carbon (%)	–	68.8	–	Phosphate solubilization	+	+

### Soil ECe

**Figure 2:**
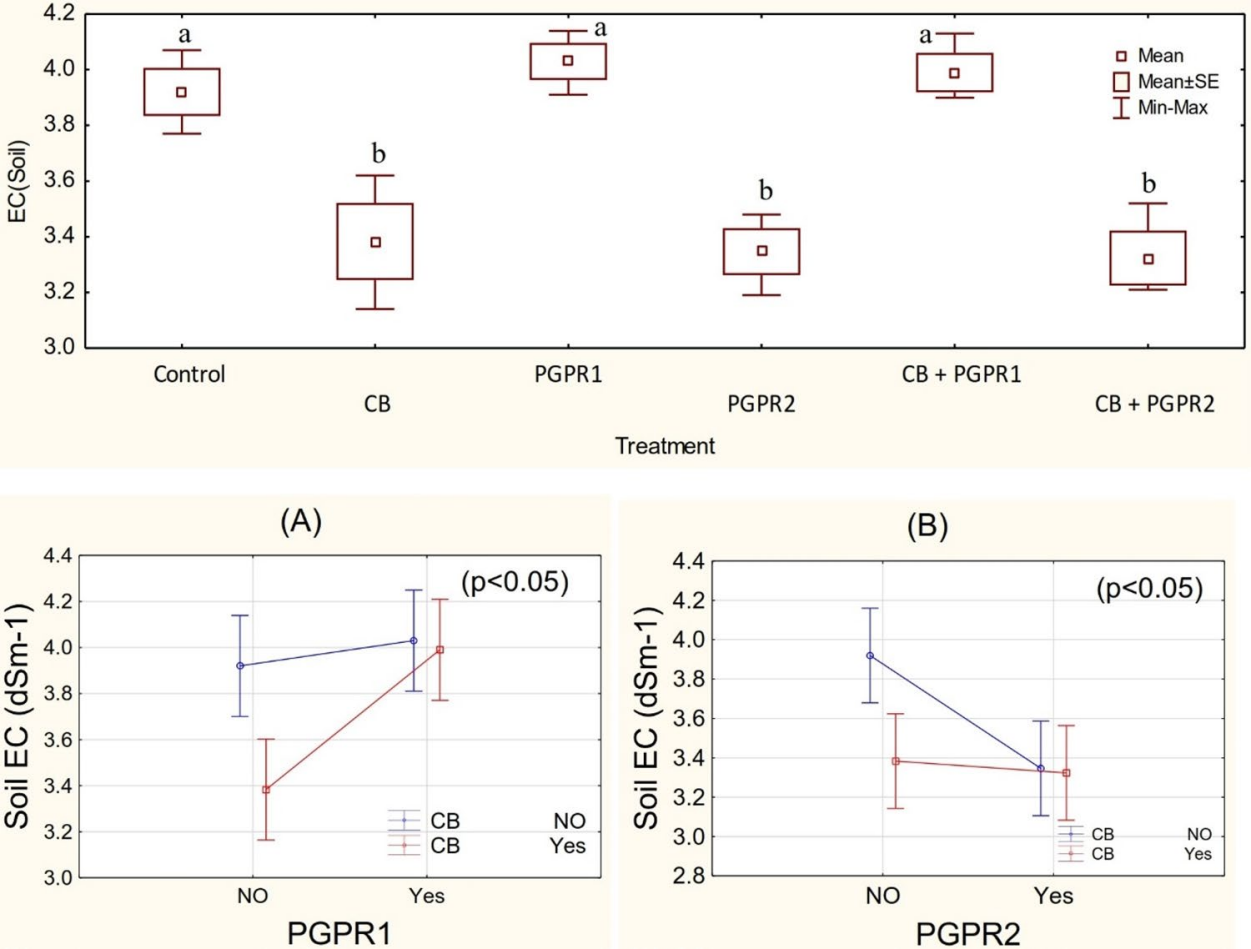
Ec*e* value (dSm^−1^) values of soil treated with CB, PGPR1, PGPR2 and their combination with CB. Means of three replicates having different small letters express significant differences at p ≤ 0.05 compared with Duncan’s test. Interaction graph of PGPR1 and CB **(A)**; PGPR2 and CB **(B)** for soil EC*e* (dSm^−1^).

One-way ANOVA showed that different treatments remained significant (p ≤ 0.05) for the decrease in soil EC*e* value over control. The result shows that PGPR1 and CB have a significant (p ≤ 0.05) main effect on soil EC (Fig. 2), and a significant ordinal interaction was found between PGPR1 and CB (Fig. 2A). Similarly, PGPR2 and CB show a significant main effect on the soil EC*e* (Fig. 2), with ordinal interaction (Fig. 2B). Addition of CB remained significant regarding the reduction in soil EC*e* over control. Inoculation of PGPR2 also remained significant from control for the decrease in soil EC*e*. It was noted CB + PGPR2 remained statistically alike with PGPR2 but differed significantly from control. For reduction in soil EC*e*, PGPR2 was significantly different as compared to PGPR1 (Fig. 2). No significant change was noted over control in the soil EC*e* where PGPR1 and CB + PGPR1 were applied. However, a maximum decrease of 17.6% in the soil EC*e* was noted from control where CB + PGPR2 was applied as an amendment. The results of the current study contrary to the other documented results regarding biochar and soil EC*e*. The reduction in the soil EC*e* in the current study might be due to high oxidation of biochar when applied by mixing in compost. Inoculation of PGPR2 might also speed up the oxidation of CB. Application of biochar significantly increases soil cation exchangeability^39^. Higher cation exchange capacity (CEC) increases the accumulation of ions in the rhizosphere that increases the soil EC*e*. However, for improvement in the soil CEC slow oxidation of biochar is a necessity^40^. In addition to the above, growth promoting PGPR increases root surface area. This improvement in the surface area of roots facilitates the plants for the uptake of nutrients^41^.

### Soil organic matter (OM)

**Figure 3:**
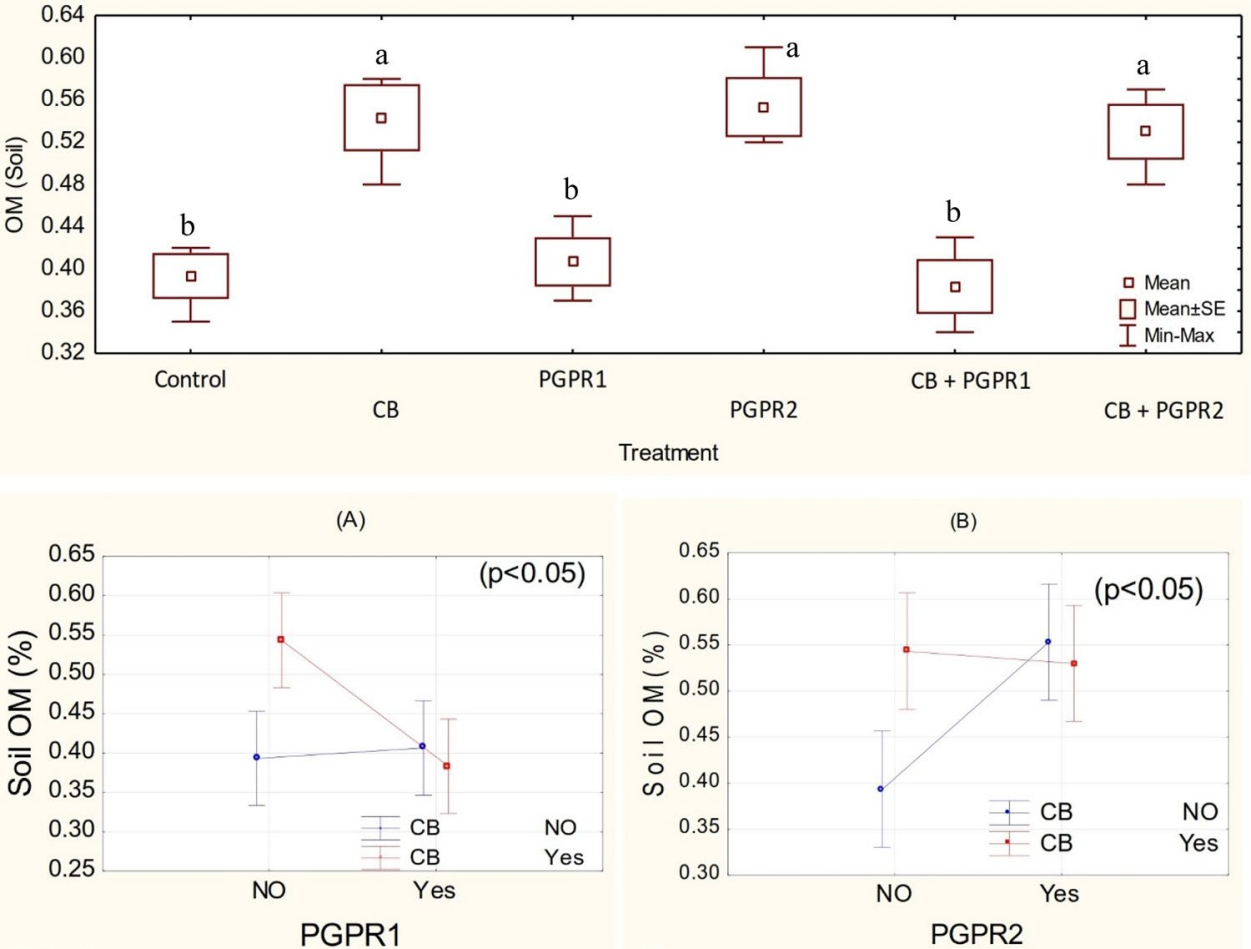
OM (%) values of soil treated with CB, PGPR1, PGPR2 and their combination with CB. Means of three replicates having different small letters express significant differences at p ≤ 0.05 compared with Duncan’s test. Interaction graph of PGPR1 and CB **(A)**; PGPR2 and CB **(B)**, for soil OM (%).

Application of different treatments remained significant (p ≤ 0.05) for an increase in OM value over control. Inoculation of PGPR and CB have significant interaction on soil OM (Fig. 3). Significant ordinal interaction was found between CB and PGPR2 (Fig. 3A). Furthermore, PGPR1 and CB also have a significant (p ≤ 0.05) ordinal interaction for soil OM (Fig. 3B). Application of CB significantly enhanced the organic matter in the soil over control. Inoculation of PGPR2 also performed significantly better than control in improving the soil OM. It was noted CB + PGPR2 also remained statistically alike with PGPR2 but differed significantly from control for the improvement in soil OM (Fig. 3). Inoculation of PGPR1 and addition of CB + PGPR1 did not differ significantly for the soil OM over control. Maximum decrease of 41.2% in soil OM was noted from control where PGPR2 was inoculated as an amendment. The improvement in the soil OM was due to better proliferation of PGPR2 and high organic carbon contents of compost mixed biochar (Table 1). Low level of OM in PGPR1 inoculated soil might be due to poor proliferation of PGPR1. Biochar is an activated form of carbon. It is produced at high temperature and limited or no oxygen that causes carbon sequestration ^42^. An application of biochar increases the soil aggregation that plays an important role in the soil OM buildup^43–45^. Furthermore, biochar indirectly promotes the soil microbial activities, biomass and growth^46,47^. In addition to the above use of compost was another important factor for enhancing the soil organic matter. Recently it has been documented that the application of organic amendments i.e., compost organic matters significantly affects the soil organic on a long term basis^48^.

### Soil N, P and K concentration

**Figure 4:**
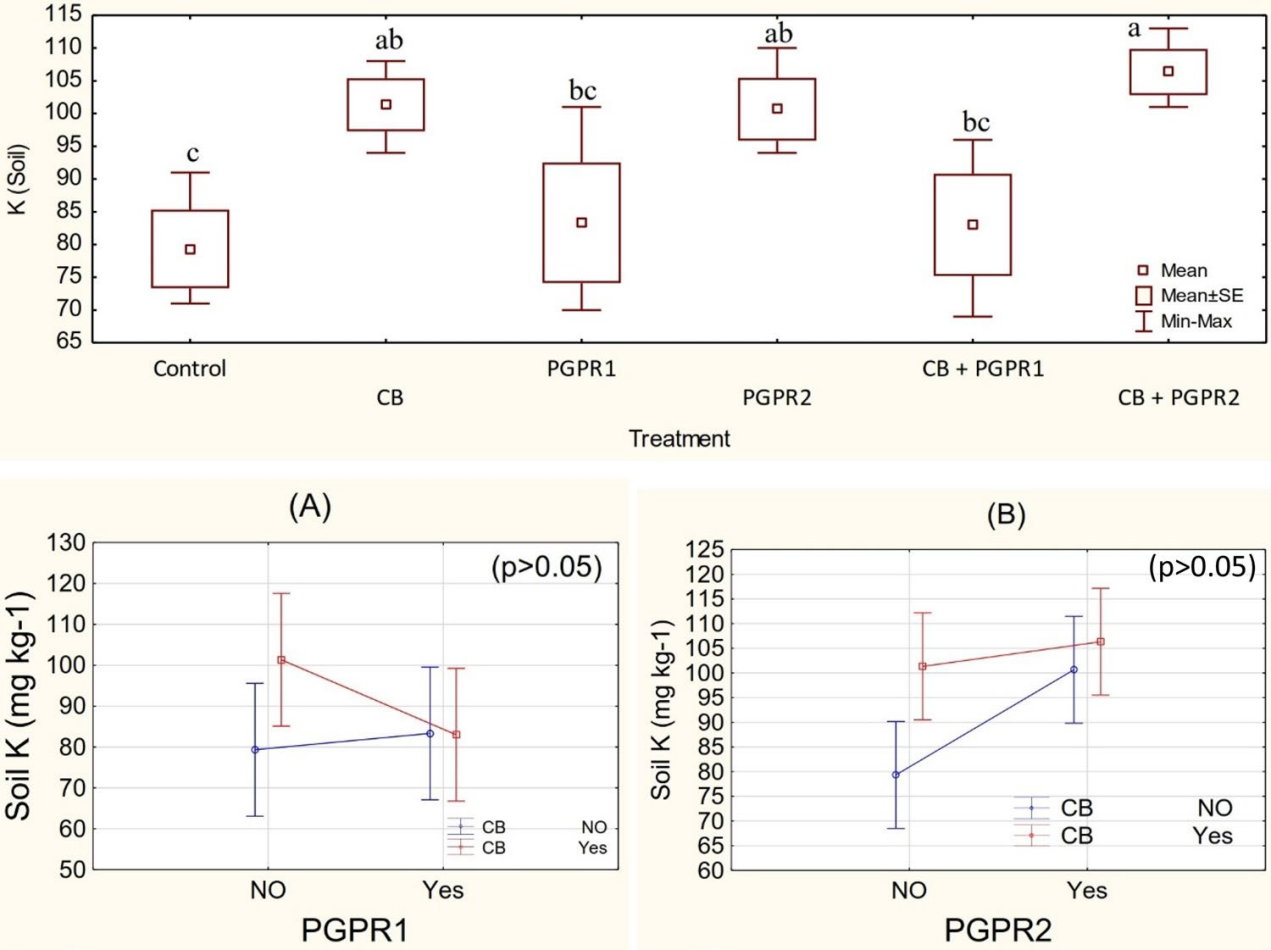
Soil K (%) values of soil treated with CB, PGPR1, PGPR2 and their combination with CB. Means of three replicates having different small letters express significant differences at p ≤ 0.05 compared with Duncan’s test. Interaction graph of PGPR1 and CB **(A)**; PGPR2 and CB **(B)**, for soil K (mg kg^−1^).

One-way ANOVA showed that treatments remained non-significant in improving the soil N (Figs. S1, S2) and P (Figs. S3, S4) contents but significant for K. It was noted that main effect of PGPR and CB (Fig. 4) were also significant for K concentration. However, non-significant ordinal interaction was found between PGPR1 and CB (Fig. 4A) as well as PGPR2 and CB (Fig. 4B). The application of CB + PGPR2 remained significant for improvement in soil K concentration over control. No significant change was observed among CB + PGPR2 and PGPR2; however, PGPR2 also differed significantly as compared to control for K concentration in the soil. The addition of CB in soil was also significantly different from control for K concentration in the soil (Fig. 4). No significant change was observed among control, PGPR1 and CB + PGPR1 for K concentration in the soil. The maximum increase of 34% in K concentration of the soil was noted where CB + PGPR2 was applied as compared to control. The increase in K concentration of soil was due to the presence of K in compost and biochar. In sole inoculation of PGPR2, solubilization of K by organic secretions might be the major cause of a significant increase in soil K concentration. Application of biochar decreases the leaching losses of nutrients^49^. High surface area and ion exchangeability of biochar make it most suitable amendment for improving the soil fertility status^50,51^. Furthermore, organic acids i.e., oxalic acid, tartaric acid, citric acid, malic acid, and succinic acid secretions of PGPR decrease the soil pH and chelate K by producing siderophores that increases its bioavailability to the plants^52,53^. Compost is enriched with the mineralized form of K which is water extractable and governs the soil fertility status^54^. Improvement in the root physiology by the application of organic manure combined with biochar also improves the nutrient’s availability to the plants^55^.

### Root and leaves fresh and dry weight

**Figure 5:**
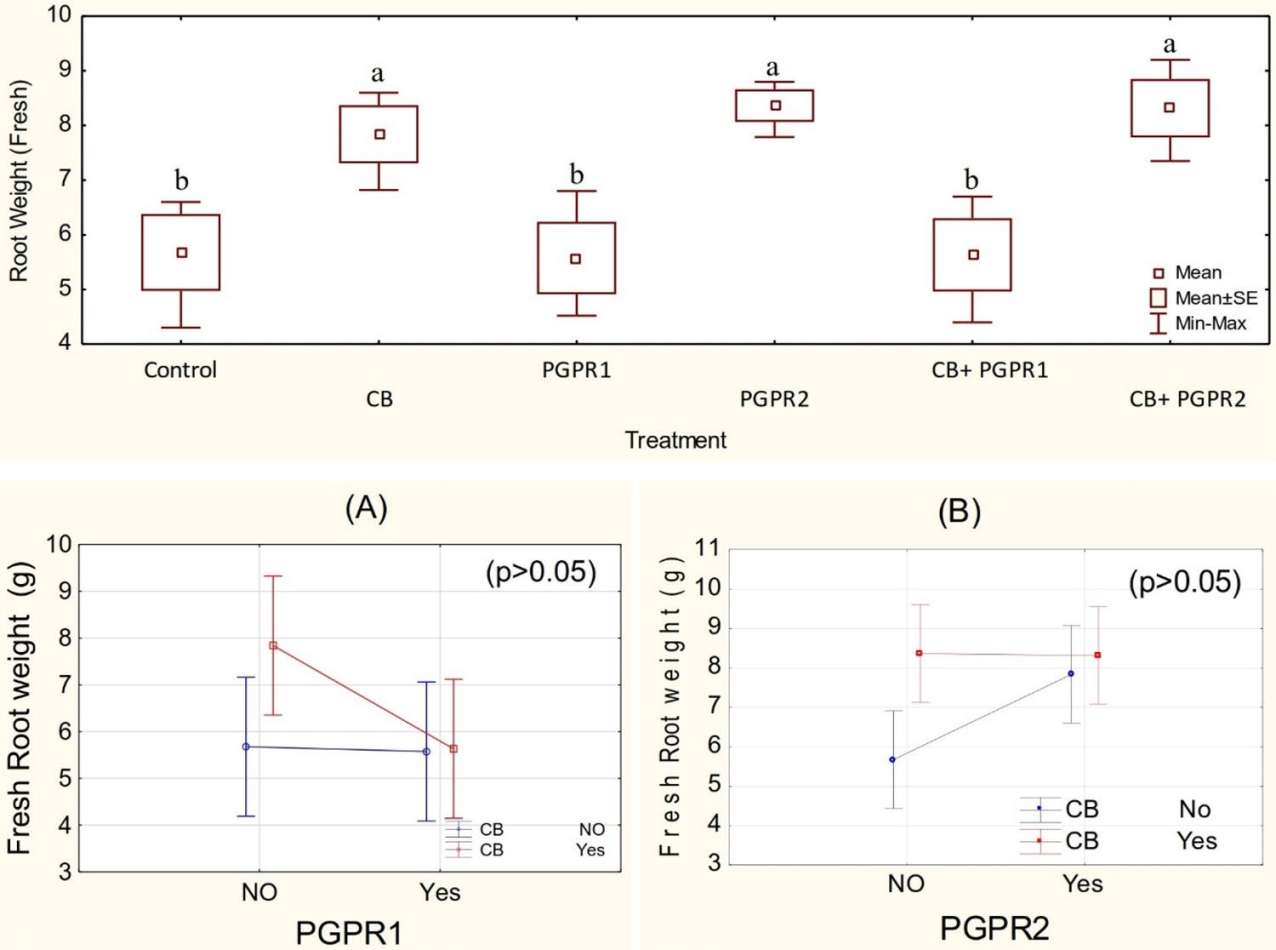
Fresh root weight (g) values of spinach treated with CB, PGPR1, PGPR2 and their combination with CB. Means of three replicates having different small letters express significant differences at p ≤ 0.05 compared with Duncan’s test. Interaction graph of PGPR1 and CB **(A)**; PGPR2 and CB **(B)**, for fresh root weight (g) of spinach plant.

**Figure 6:**
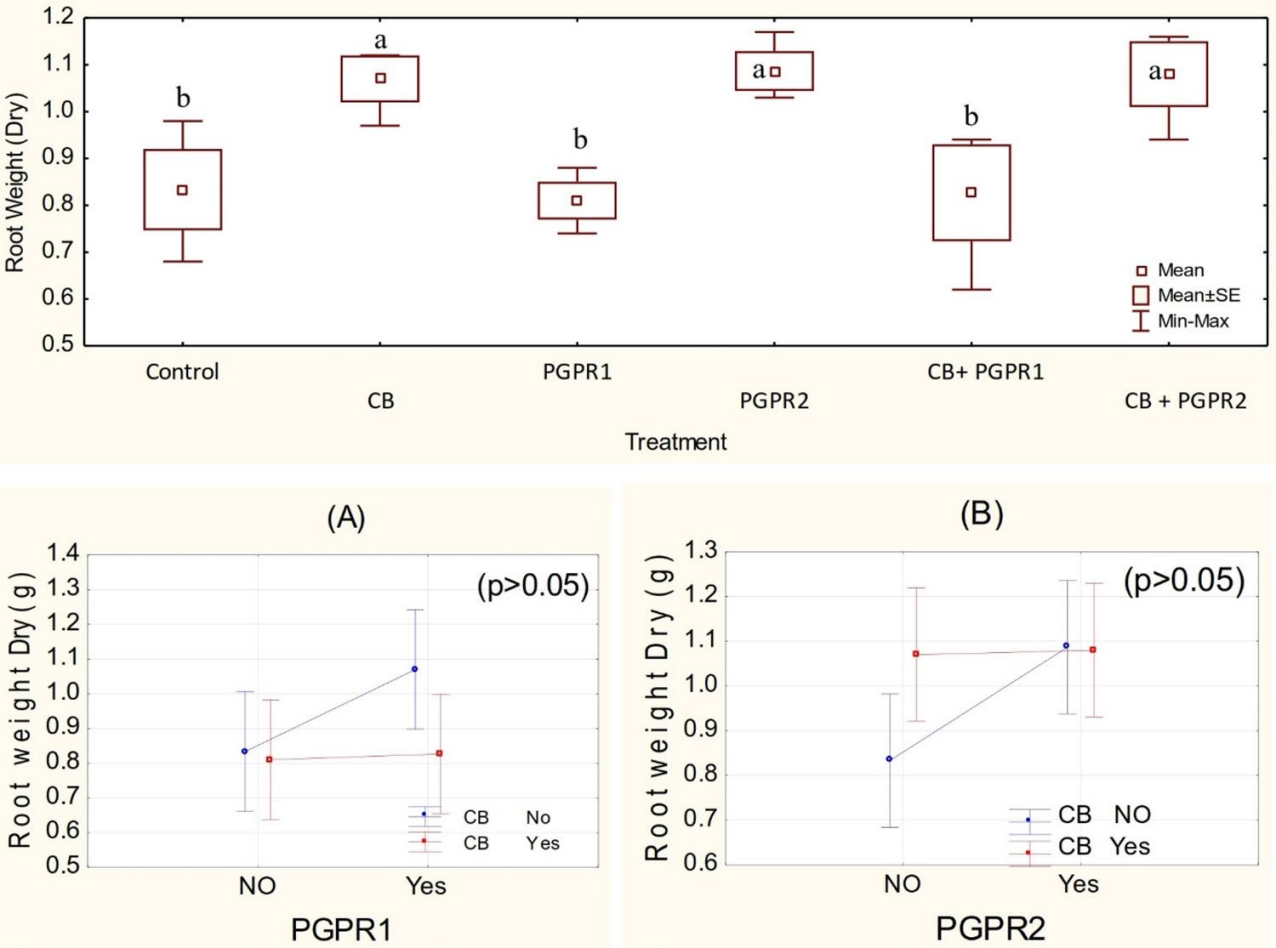
Dry root weight (g) values of spinach treated with CB, PGPR1, PGPR2 and their combination with CB. Means of three replicates having different small letters express significant differences at p ≤ 0.05 compared with Duncan’s test. Interaction graph of PGPR1 and CB **(A)**; PGPR2 and CB **(B)**, for dry root weight (g) of spinach plant.

One way ANOVA showed that treatments were non-significant for leaves fresh (Figs. S5 and S6) and dry (Figs. S7, S8) weight but significant for root fresh (Fig. 5) and dry (Fig. 6) weight under Pb stress. Significant (p ≤ 0.05) main effect (Fig. 5) but non-significant ordinal interaction was observed between PGPR1 (Figs. 5A and 6A) and PGPR2 (Figs. 5B and 6B) with CB for root fresh and dry weight respectively. It was noted that CB + PGPR2 differed significantly for root fresh and dry weight over control. No significant change was noted among CB, CB + PGPR2 and PGPR2; however, PGPR2 also differed significantly as compared to control for the root fresh and dry weight. The addition of CB in the soil was also significantly different from control for improvement in root fresh and dry weight under Pb stress. Furthermore, the sole inoculation of PGPR2 was significantly different for improving root fresh and dry weight from PGPR1 (Figs. 5 and 6). No significant change was noted from control where PGPR1 and CB + PGPR1 were applied for root fresh and dry weight. The maximum increase of 47 and 31% in the root fresh and dry weight was noted where PGPR2 was inoculated as compared to control, respectively. Lead is one of the heavy metal pollutants which is not essential for the plant growth and remains accumulated in the roots. The higher amount of Pb decreases the root growth, thus induces negative effects on the plants. In the current study, increase in the dry and fresh weight of the leaves and roots of the spinach crops might be due to the siderophores production, phosphate solubilization and by providing the systematic resistance against heavy metal stress through plant growth-promoting rhizobacteria^56,57^. Secretion of indole acetic acid by PGPR improves the elongation of roots^58^. According to Mohite^59^ IAA promotes the growth of adventitious roots that play an important role in the uptake of nutrients. Biochar and compost application increase the plant biomass production due to the improvement in the uptake of nutrients in the plants and/or soil physicochemical properties^60,61^. The high porosity of biochar makes it an important organic amendment that decreases the losses of nutrients^62^.

### N, P and K concentration in plant

**Figure 7:**
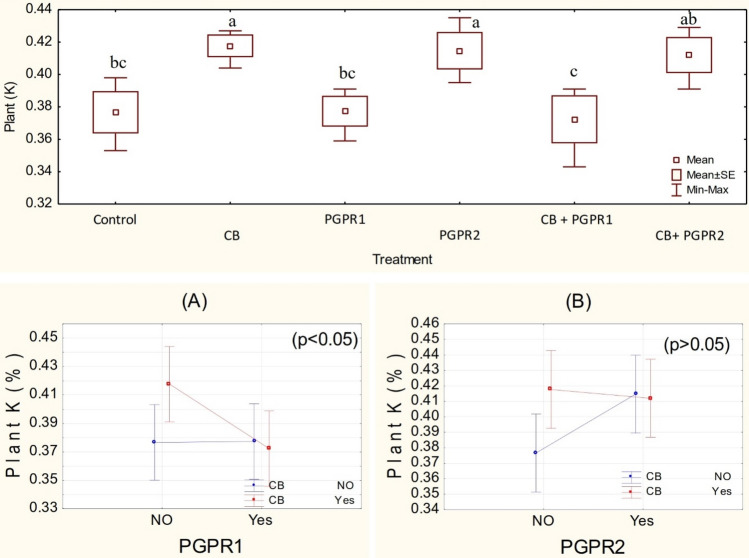
Plant K value (%) values treated with CB, PGPR1, PGPR2 and their combination with CB. Means of three replicates having different small letters express significant differences at p ≤ 0.05 compared with Duncan’s test. Interaction graph of PGPR1 and CB **(A)**; PGPR2 and CB **(B)**, for plant K value (%).

One-way ANOVA showed that treatments did not differ significantly for N (Figs. S9, S10) and P (Figs. S11, S12) concentration but remained significant (p ≤ 0.05) for K concentration in the plants. Inoculation of PGPR1 showed significant main effect whereas CB does not have a significant main effect, although there was a significant interaction between CB and PGPR1 (Fig. 7A). It was observed that PGPR2 showed non-significant while PGPR1 showed significant, ordinal interaction with CB (Fig. 7A,B). Application of CB and PGPR2 differed significantly for K concentration in the plants over control. No significant change was noted among CB, CB + PGPR2 and PGPR2; however, CB + PGPR2 and PGPR2 also differed significantly as compared to control for K concentration in the plant (Fig. 7). In addition, treatment CB + PGPR2 remained significant as compared to CB + PGPR1 for K concentration in the plants. No significant change was noted from control where PGPR1 and CB + PGPR1 were applied for K concentration in the plants. The maximum increase of 10.5% in K concentration was observed where CB was inoculated as compared to control, respectively. The improvement in K concentration of the plant might be due to the plant growth promoting rhizobacteria which promoted the uptake and availability of the nutrients by recycling^63^, solubilization^64^ of nutrients and siderophores production^56^. Besides, the imperative role of plant growth-promoting rhizobacteria, the BC, has a high water holding capacity, ion exchange capacity, high surface area that make it an effective amendment for enhanced uptake of water and nutrients in the plants^65–67^. Depending upon the feedstock, biochar itself carries a significant amount of mineral nutrients^68^. The application of compost also increased the nutrient status in the soils which consequently increased the uptake of nutrient in the crops^61^. According to Schulz et al.^69^, composted biochar significantly increased total organic carbon (TOC) that plays an imperative role in the uptake of nutrients. Danish and Zafar-ul-Hye^24^ suggested that the performance of PGPR for nutrients uptake in the crops can be enhanced when they are applied in combination with timber waste biochar^70^.

### Chlorophyll and Pb concentration in plant

**Figure 8:**
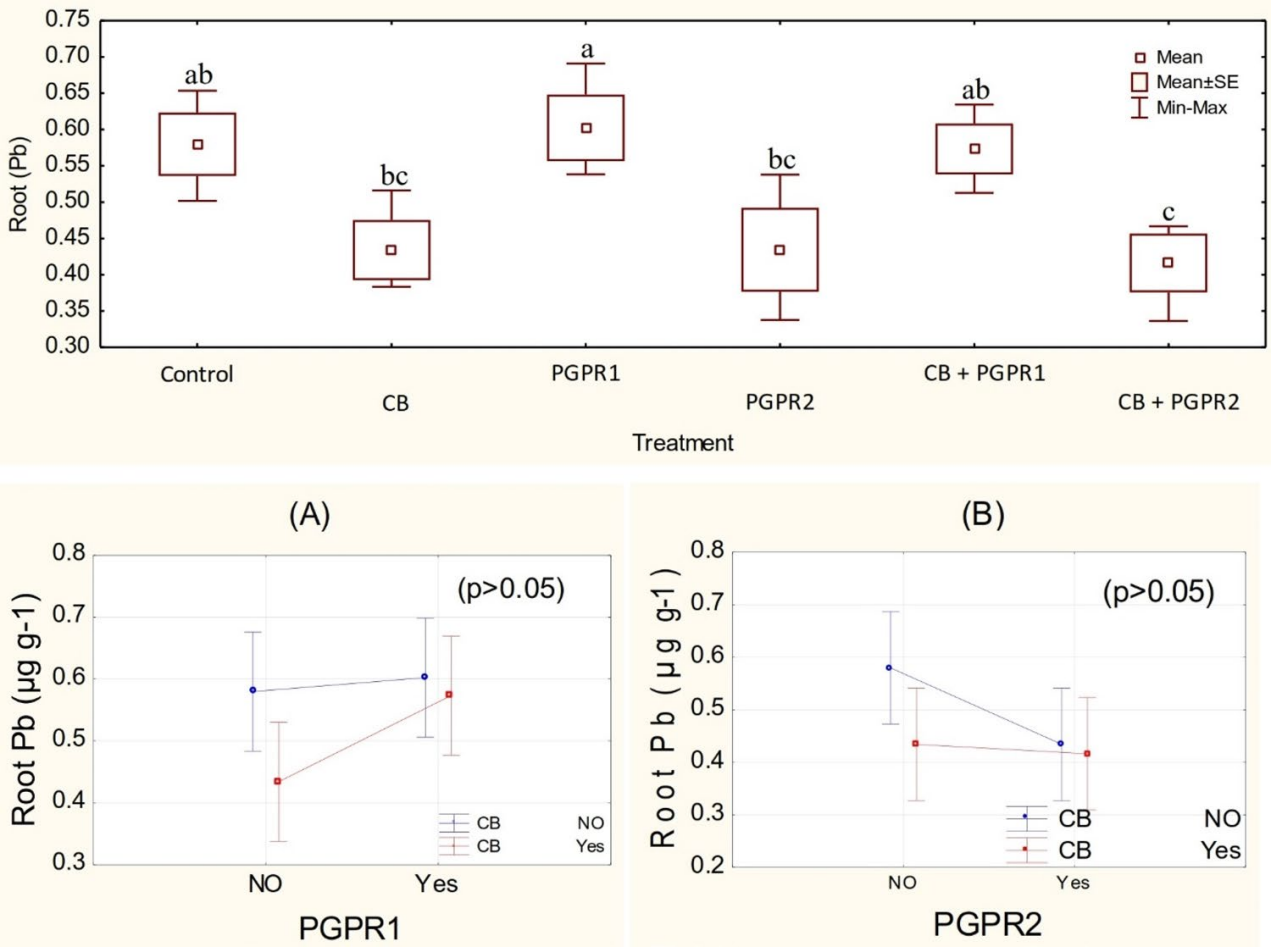
Root Pb value (µg g^−1^) of spinach treated with CB, PGPR1, PGPR2 and their combination with CB. Means of three replicates having different small letters express significant differences at p ≤ 0.05 compared with Duncan’s test. Interaction graph of PGPR1 and CB **(A)**; PGPR2 and CB **(B)**, for plant K value (%) for Root Pb value (µg g^−1^) of spinach plant.

One-way ANOVA showed that treatments did not differ significantly for chlorophyll contents (Figs. S13, S14) and Pb concentration in leaves (Figs. S15, S16). However, the effect of treatments was significant (p ≤ 0.05) for Pb concentration in the roots of plants (Fig. 8). It was observed that PGPR and CB do not have significant interaction whereas PGPR show significant main effect (Fig. 8A) for Pb concentration in roots. Sole application of CB + PGPR2 differed significantly for less uptake of Pb in the roots as compared to control. No significant change was noted among CB and PGPR2; however, CB and PGPR2 also differed significantly better as compared to control for less uptake of Pb by the plants’ roots. Inoculation of PGPR2 was significant as compared to PGPR1 for decreasing Pb concentration in the plant’s roots (Fig. 8). However, PGPR1 and CB + PGPR1 did not differ significantly as compared to control for Pb concentration in the roots. Maximum decrease of 43% in Pb concentration was noted where CB was inoculated as compared to control, respectively. Under heavy metal toxicity, production of ethylene is significantly increased in the roots that induced adverse effects on the growth of plants. Production of ACC deaminase breaks this endogenous stress generating ethylene into α-ketobutyrate and ammonia that mitigate heavy metal stress ^71,72^. Similar, kind of improvement was also noted by Zafar-ul-Hye et al.^73^ when they inoculated wheat while ACC deaminase was producing PGPR under toxicity of heavy metal. Furthermore, application of compost provided energy to rhizobacteria and improved the transfer of oxygen which played an important role in the immobilization of metallic ions in the soil^74^. Song and Greenway^75^ argued that binding of heavy metals with exchange sites reduced their bioavailability to plants. Active function groups on the surface of biochar adsorb heavy metals electrostatically, causing their immobilization in the soil through cation exchange mechanism^76^. Presence of CO_3_^−2^ and hydroxides on biochar surface also played an imperative role in the immobilization of divalent heavy metals^77,78^. Through organic chelating agents and secretions, PGPR changes the redox potential in the rhizosphere. Change in redox potential and acidification of rhizosphere by PGPR decreases the bioavailability of heavy metals to the plants^79,80^.

## Conclusion

It is concluded that the application of PGPR2 i.e., *Bacillus amyloliquefaciens* with compost mixed biochar can alleviate the Pb toxicity by the improving the nutrients uptake The combined use of ACC deaminase producing PGPR2 *Bacillus amyloliquefaciens* and compost mix biochar can improve the *Spinacia oleracea* L. root growth and K uptake in the plants under Pb stress. However, more investigation is needed at a field level to introduce combined use of *Bacillus amyloliquefaciens* and compost mix biochar as an efficacious amendment against mitigation of Pb toxicity in the crops.

## Materials and methodology

For the production of biochar, the waste material of vegetables and fruits were collected from the SabziMandi, Multan. These waste materials were air-dried under suitable sunlight for two weeks until the moisture content remained < 15%. The waste material was chopped into small pieces, then filled in the electric pyrolyzer and heated at 450° C temperature for 120 min under anaerobic condition. The pyrolyzer was allowed to cool down at an average temperature. The prepared biochar sample was removed from the pyrolyzer, grinded and further allowed to pass through 2 mm sieve. Biochar was applied at 0.5% (5 g kg^−1^) soil according to the treatment plan in the pots. The Prepared compost, manufactured by Buraq Agro Chemicals, Industrial State Area, Multan was applied at 0.5% (5 g kg^−1^) soil according to the treatment plan. Two rhizobacterial strains previously identified as PGPR1 *Alcaligenes faecalis* and PGPR2 *Bacillus amyloliquefaciens* were obtained from the Soil Microbiology and Biochemistry Laboratory, BZU, Multan. The respective inoculum of rhizobacteria was prepared in Dworkin and Foster (DF) media present in 250 ml Erlenmeyer flasks^81^. Each flask containing DF media was inoculated with respective strains for 72 h at the laboratory temperature. The spinach seeds were inoculated (inoculum density 0.5 nm) with respective bacterial inoculum (5 ml 100 g^−1^ seeds) and mixed with sterilized clay, peat and sugar solution at the time of sowing.

A pot experiment was conducted in the warehouse at the experimental farm of the Faculty of Agricultural Sciences and Technology, Bahauddin Zakariya University, Multan. The effect of biochar, compost and plant growth-promoting rhizobacteria was evaluated by designing the experiment on the spinach grown on lead-contaminated soil. Six treatments were arranged with three replications in Complete Randomized Design (CRD). The pre-experimental soil characteristics are provided in Table 1. Each pot was filled with 7 kg of soil. A basal dose of K_2_O, P_2_O_5_ and N, was applied at the rate of 130, 90 and 110 kg per hectare, in the form of SOP, DAP and urea, respectively for the spinach crop^82^. The total of phosphate and potash fertilizer were applied at the time of sowing while N was applied in three splits. After two weeks of germination, Pb stress was applied artificially. Lead sulphate (PbSO_4_) was applied for introducing 250 mg Pb kg^−1^ soil^83^.There were six treatments as i.e. control, PGPR1 (*Alcaligenes faecalis*), PGPR2 (*Bacillus amyloliquefaciens*), compost + biochar (CB) (1:1), CB + PGPR1 and CB + PGPR2.

Bouyoucos hydrometer method was applied for sand, silt and clay percentage determination^84^. Soil saturated paste was prepared by adding distilled water in a plastic beaker containing 300 g of soil sample. The pH meter was calibrated using a buffer solution of strength 4, 7 and 9.2 pH. Then the pH of saturated paste was determined on it. Already prepared soil saturated paste for pH was extracted by a vacuum pump to get a clear extract. Electrical conductivity (EC) meter was calibrated with KCl solution (0.01 N), and EC*e* of the sample was measured in dS m^−1^. Walkley–Black^85^ method was used to measure the organic matter in the soil. Kjeldahl’s distillation method was used to measure the total N in the soil. For that H_2_SO_4_with digestion mixture (FeSO_4_: K_2_SO_4_: CuSO_4_, 1:10:5) was used for digestion. The evolved NH_3_ was absorbed in boric acid solution in a receiver having methyl red and bromocresol green indicators. The content was titrated with H_2_SO_4,_ and N was calculated in percentage^86^. Sodium bicarbonate solution was used to extract 5 g soil sample by shaking on a mechanical shaker. 8 ml of colour developing reagent and 2 ml of aliquot was taken in 50 ml flask. Extractable soil phosphorus was measured at 880 nm wavelength with spectrophotometer^87^. Ammonium acetate method was used to extract soil potassium. The extractable soil potassium was calculated by using a flame photometer. In a 50 ml conical flask 10 g soil sample was taken. The soil sample was extracted with 20 ml of 0.01 M CaCl_2_ + 0.01 M TEA + 0.005 M DTPA solution. Extracting solution pH was adjusted up to 7.3 and shook for 120 min^88^. The atomic absorption spectrophotometer was used for measuring the Pb concentration^89^.

The samples were weighted two weeks later when they had been dried up by an electrical balance. Nitrogen was analyzed by using 2 ml of digested plant sample, 1 ml of 0.17 mm Na nitroprusside in 1% (w/v) phenol, 1.0 ml of a solution containing 0.125 N NaOH, 0.25 M Na_2_HPO_4_ in 0.03% (w/v) NaOCl. Test tube containing the above solution was mixed vigorously on a vortex mixer and incubated in a water bath at 37 °C for 30 min. The absorbance was measured at 625 nm on a spectrophotometer. For determination of phosphorus, the plant samples were digested in an acid mixture of HNO_3_ and HCIO_4_^90^. The ammonium vanadate and ammonium molybdatewere added as colour developing reagents in the aliquot. The phosphorus was determined at 470 nm wavelength by using spectrophotometer after calibrating with P standards^91^. For determination of potassium, the digested sample aliquot was fed to the flamephotometer^92^. The reading of filtrate and standards was noted on atomic absorption spectrophotometer.

Data were analyzed by following standard statistical procedure ^93^. One way and two-way ANOVA were applied by using SPSS 20. Treatments were compared using Duncan's test for differentiation at p ≤ 0.05.

## Supplementary information

Supplementary Figures

## Data Availability

No datasets were generated or analyzed during the current study. All the analyzed data can be accessed after publication by requesting the corresponding author.

## References

[1] Adrees M (2015). Mechanisms of silicon-mediated alleviation of heavy metal toxicity in plants: A review. Ecotoxicol. Environ. Saf..

[2] Keller, C. *et al.* Effect of silicon on wheat seedlings (*Triticum turgidum* L.) grown in hydroponics and exposed to 0 to 30 µM Cu. *Planta* **241**, 847–860 (2015). 10.1007/s00425-014-2220-1 25515193

[3] Adriano. Trace elements in terrestrial environments. In *Biogeochemistry, Bioavailability and Risks of Metals *Vol. 32 374. (Springer, New York, 2001).

[4] Seregin IV, Kozhevnikova AD (2008). Roles of root and shoot tissues in transport and accumulation of cadmium, lead, nickel, and strontium. Russ. J. Plant Physiol..

[5] Hall JL (2002). Cellular mechanisms for heavy metal detoxification and tolerance. J. Exp. Bot..

[6] Saleem M, Asghar HN, Zahir ZA, Shahid M (2018). Impact of lead tolerant plant growth promoting rhizobacteria on growth, physiology, antioxidant activities, yield and lead content in sunflower in lead contaminated soil. Chemosphere.

[7] Wierzbicka MH (2007). Comparison of the toxicity and distribution of cadmium and lead in plant cells. Protoplasma.

[8] Xiong, Z. T., Zhao, F. & Li, M. J. Lead toxicity in Brassica pekinensis Rupr.: Effect on nitrate assimilation and growth. *Environ. Toxicol.* **21**, 147–153 (2006). 10.1002/tox.20167 16528690

[9] Zulfiqar U (2019). Lead toxicity in plants: Impacts and remediation. J. Environ. Manag..

[10] Sharma P, Dubey RS (2005). Lead toxicity in plants. Brazilian J. Plant Physiol..

[11] Broyer TC, Johnson CM, Paull RE (1972). Some aspects of lead in plant nutrition. Plant Soil.

[12] Ahmad M (2016). Lead and copper immobilization in a shooting range soil using soybean stover- and pine needle-derived biochars: Chemical, microbial and spectroscopic assessments. J. Hazard. Mater..

[13] Rizwan M (2016). Mechanisms of biochar-mediated alleviation of toxicity oftrace elements in plants: a critical review. Environ. Sci. Pollut. Res..

[14] Li H (2016). Biochar amendment immobilizes lead in rice paddy soils and reduces its phytoavailability. Sci. Rep..

[15] Gao J (2020). A promising and cost-effective biochar adsorbent derived from jujube pit for the removal of Pb(II) from aqueous solution. Sci. Rep..

[16] Liu L, Deng G, Shi X (2020). Adsorption characteristics and mechanism of p-nitrophenol by pine sawdust biochar samples produced at different pyrolysis temperatures. Sci. Rep..

[17] Bolan NS, Duraisamy VP (2003). Role of inorganic and organic soil amendments on immobilisation and phytoavailability of heavy metals: A review involving specific case studies. Aust. J. Soil Res..

[18] Jiang J, Xu R, Jiang T, Li Z (2012). Immobilization of Cu(II), Pb(II) and Cd(II) by the addition of rice straw derived biochar to a simulated polluted Ultisol. J. Hazard. Mater..

[19] Schulz H, Dunst G, Glaser B (2014). No effect level of co-composted biochar on plant growth and soil properties in a greenhouse experiment. Agronomy.

[20] Van Loon LC (2007). Plant responses to plant growth-promoting rhizobacteria. Eur. J. Plant Pathol..

[21] Bakker PAHM, Pieterse CMJ, Van Loon LC (2007). Induced systemic resistance by fluorescent *Pseudomonas* spp. Phytopathology.

[22] Danish S (2015). Phosphorus solubilizing bacteria and rice straw biochar consequence on maize pigments synthesis. Int. J. Biosci..

[23] Danish S, Zafar-ul-Hye M, Hussain M, Shaaban M, Núñez-delgado A (2019). Rhizobacteria with ACC-deaminase activity improve nutrient uptake, chlorophyll contents and early seedling growth of wheat under peg- induced osmotic stress. Int. J. Agric. Biol..

[24] Danish S, Zafar-ul-Hye M (2019). Co-application of ACC-deaminase producing PGPR and timber-waste biochar improves pigments formation, growth and yield of wheat under drought stress. Sci. Rep..

[25] Danish S (2019). Alleviation of chromium toxicity in maize by Fe fortification and chromium tolerant ACC deaminase producing plant growth promoting rhizobacteria. Ecotoxicol. Environ. Saf..

[26] Danish, S. & Zafar-ul-Hye, M. Combined role of ACC deaminase producing bacteria and biochar on cereals productivity under drought. *Phyton (B. Aires).* **89**, 217–227 (2020).

[27] Zafar-ul-Hye, M. *et al.* Multi-strain inoculation with pgpr producing acc deaminase is more effective than single-strain inoculation to improve wheat (*Triticum aestivum*) growth and yield. *Phyton (B. Aires).* **89**, 405–413 (2020).

[28] Danish S, Zafar-ul-Hye M, Mohsin F, Id MH (2020). ACC-deaminase producing plant growth promoting rhizobacteria and biochar mitigate adverse effects of drought stress on maize growth. PLoS ONE.

[29] Liu, W., Du, L. & Yang, Q. Biogas slurry added amino acids decreased nitrate concentrations of lettuce in sand culture. *Acta Agric. Scand. Sect. B Soil Plant Sci.* **59**, 260–264 (2009).

[30] Figueiredo, M. V. B., Martinez, C. R., Burity, H. A. & Chanway, C. P. Plant growth-promoting rhizobacteria for improving nodulation and nitrogen fixation in the common bean (*Phaseolus vulgaris* L.). *World J. Microbiol. Biotechnol.* **24**, 1187–1193 (2008).

[31] Lamhamdi M (2013). Effect of lead stress on mineral content and growth of wheat (*Triticum aestivum*) and spinach (*Spinacia oleracea*) seedlings. Saudi J. Biol. Sci..

[32] Aehle E (2004). Development and evaluation of an enriched natural antioxidant preparation obtained from aqueous spinach (*Spinacia oleracea*) extracts by an adsorption procedure. Food Chem..

[33] Maeda, N., Yoshida, H. & Mizushina, Y. Spinach and health: anticancer effect. In *Bioactive Foods in Promoting Health: Fruit and Vegetables* (eds. Watson, R. R. & Preedy, V. R.) 393–405 (Elsevier, London, 2010).

[34] Rizwan ST, Chaudhary S, Ikram M (2013). Uptake of some toxic metals in spinach crop irrigated by Saggian drain water, Lahore. Biology.

[35] Etesami, H. & Adl, S. M. Plant growth-promoting rhizobacteria (PGPR) and their action mechanisms in availability of nutrients to plants. In *Phyto-Microbiome in Stress Regulation* (eds. Kumar, M., Kumar, V. & Prasad, R.) 147–203 (Springer, Singapore, 2020). 10.1007/978-981-15-2576-6_9.

[36] Nardi, S., Carletti, P., Pizzeghello, D. & Muscolo, A. Biological activities of humic substances, in biophysicochemical processes involving natural nonliving organic matter in environmental systems. In *Fundamentals and Impact of Mineral-Organic-Biota Interactions on the Formation, Transformation, Turnover, and Storage of Natural Nonliving Organic Matter (NOM)* (eds. Senesi, N., Xing, B. & Huang, P. M.) (John Wiley, New York, 2009).

[37] Li M (2019). Population characteristics and influential factors of nitrogen cycling functional genes in heavy metal contaminated soil remediated by biochar and compost. Sci. Total Environ..

[38] Zhang J (2016). Ammonia-oxidizing bacterial communities and shaping factors with different: Phanerochaete chrysosporium inoculation regimes during agricultural waste composting. RSC Adv..

[39] Laird D, Fleming P, Wang B, Horton R, Karlen D (2010). Biochar impact on nutrient leaching from a Midwestern agricultural soil. Geoderma.

[40] Brodowski S, Amelung W, Haumaier L, Abetz C, Zech W (2005). Morphological and chemical properties of black carbon in physical soil fractions as revealed by scanning electron microscopy and energy-dispersive X-ray spectroscopy. Geoderma.

[41] Egamberdieva, D., Davranov, K., Wirth, S., Hashem, A. & Abd_Allah, E. F. Impact of soil salinity on the plant-growth—Promoting and biological control abilities of root associated bacteria. *Saudi J. Biol. Sci.* **24**, 1601–1608 (2017). 10.1016/j.sjbs.2017.07.004 PMC5643845 29062259

[42] Thies, J. & Rillig, M. C. Characteristics of biochar: Biological properties. In* Biochar for Environmental Management: Science and Technology*. (2009).

[43] Spokas KA, Koskinen WC, Baker JM, Reicosky DC (2009). Impacts of woodchip biochar additions on greenhouse gas production and sorption/degradation of two herbicides in a Minnesota soil. Chemosphere.

[44] Cao X, Ma L, Gao B, Harris W (2009). Dairy-manure derived biochar effectively sorbs lead and atrazine. Environ. Sci. Technol..

[45] Zhang A (2010). Effect of biochar amendment on yield and methane and nitrous oxide emissions from a rice paddy from Tai Lake plain, China. Agric. Ecosyst. Environ..

[46] O’Neill B (2009). Bacterial community composition in Brazilian Anthrosols and adjacent soils characterized using culturing and molecular identification. Microb. Ecol..

[47] Joseph S (2015). Effects of enriched biochars containing magnetic iron nanoparticles on mycorrhizal colonisation, plant growth, nutrient uptake and soil quality improvement. Pedosphere.

[48] Musadji NY (2020). Spectral characteristics of soil dissolved organic matter: Long-term effects of exogenous organic matter on soil organic matter and spatial-temporal changes. Chemosphere.

[49] El-Naggar AH (2015). Carbon mineralization and nutrient availability in calcareous sandy soils amended with woody waste biochar. Chemosphere.

[50] Ippolito, J. a, Laird, D. a & Busscher, W. J. Environmental benefits of biochar. *J. Environ. Qual.* **41**, 967–72 (2012). 10.2134/jeq2012.0151 22751039

[51] Liang B (2006). Black carbon increases cation exchange capacity in soils. Soil Sci. Soc. Am. J..

[52] Prakash, J., Ram, V. & Meena, S. Potassium-solubilizing bacteria and their application in agriculture. In *Potassium Solubilizing Microorganisms for Sustainable Agriculture* 293–313 (Springer, New York, 2016). 10.1007/978-81-322-2776-2.

[53] Ahmad, M., Zahir, Z. A., Asghar, H. N. & Arshad, M. The combined application of rhizobial strains and plant growth promoting rhizobacteria improves growth and productivity of mung bean (*Vigna radiata* L.) under salt-stressed conditions. *Ann. Microbiol.* **62**, 1321–1330 (2012).

[54] Yermiyahu U (2004). Nitrogen, phosphorus, and potassium uptake by wheat and their distribution in soil following successive, Annual Compost Applications. J. Environ. Qual..

[55] Zhang Z, Dong X, Wang S, Pu X (2020). Benefits of organic manure combined with biochar amendments to cotton root growth and yield under continuous cropping systems in Xinjiang, China. Sci. Rep..

[56] Meyer JM (2000). Proverdines: Pigments, siderophores and potential taxonomic markers of fluorescent pseudomonas species. Arch. Microbiol..

[57] Peña, H. B. & Reyes, I. Nitrogen fixing bacteria and phosphate solubilizers isolated in lettuce (*Lactuca sativa* L.) and evaluated as plant growth promoters. *Interciencia* **32**, 560–565 (2007).

[58] Xie H, Pasternak JJ, Glick BR (1996). Isolation and characterization of mutants of the plant growth-promoting rhizobacterium *Pseudomonas putida* GR12-2 that overproduce indoleacetic acid. Curr. Microbiol..

[59] Mohite B (2013). Isolation and characterization of indole acetic acid ( IAA ) producing bacteria from rhizospheric soil and its effect on plant growth. J. Sci. Plant Nutr..

[60] Gupta, A., Meyer, J. M. & Goel, R. Development of heavy metal-resistant mutants of phosphate solubilizing *Pseudomonas* sp. NBRI 4014 and their characterization. *Curr. Microbiol.* **45**, 323–327 (2002). 10.1007/s00284-002-3762-1 12232661

[61] Younis, U. *et al.* Agr. *Environ. Sci. Pollut. Res.* **23**, 21385–21394 (2016). 10.1007/s11356-016-7344-3 27502564

[62] Ding Y (2016). Biochar to improve soil fertility. A review. Agron. Sustain. Dev..

[63] Asghar HN, Ishaq M, Zahir ZA, Khalid M, Arshad M (2006). Response of radish to integrated use of nitrogen fertilizer and recycled organic waste. Pak. J. Bot..

[64] Yasmin H, Bano A (2011). Isolation and characterization of phosphate solubilizing bacteria from rhizosphere soil of weeds of khewra salt range and attock. Pak. J. Bot..

[65] Brahmaprakash GP, Sahu PK (2012). Biofertilizers for sustainability. J. Indian Inst. Sci..

[66] Lehmann J, Gaunt J, Rondon M (2006). Bio-char sequestration in terrestrial ecosystems—A review. Mitig. Adapt. Strateg. Glob. Chang..

[67] Paetsch, L. *et al.* Effect of in-situ aged and fresh biochar on soil hydraulic conditions and microbial C use under drought conditions. *Sci. Rep.* **8** (2018). 10.1038/s41598-018-25039-x PMC5931515 29717234

[68] Qayyum MF, Abid M, Danish S, Saeed MK, Ali MA (2014). Effects of various biochars on seed germination and carbon mineralization in an alkaline soil. Pak. J. Agric. Sci..

[69] Schulz H, Dunst G, Glaser B (2013). Positive effects of composted biochar on plant growth and soil fertility. Agron. Sustain. Dev..

[70] Zafar-ul-Hye M, Danish S, Abbas M, Ahmad M, Munir TM (2019). ACC deaminase producing PGPR *Bacillus amyloliquefaciens* and agrobacterium fabrum along with biochar improve wheat productivity under drought stress. Agronomy.

[71] Burd GI, Dixon DG, Glick BR (1998). A plant growth-promoting bacterium that decreases nickel toxicity in seedlings. Appl. Environ. Microbiol..

[72] Glick BR (1995). The enhancement of plant growth by free-living bacteria. Can. J. Microbiol..

[73] Zafar-ul-Hye M, Shahjahan A, Danish S, Abid M, Qayyum MF (2018). Mitigation of cadmium toxicity induced stress in wheat by ACC-deaminase containing PGPR isolated from cadmium polluted wheat rhizosphere. Pak. J. Bot..

[74] Haritash AK, Kaushik CP (2009). Biodegradation aspects of polycyclic aromatic hydrocarbons (PAHs): A review. J. Hazard. Mater..

[75] Song QJ, Greenway GM (2004). A study of the elemental leachability and retention capability of compost. J. Environ. Monit..

[76] Yuan JH, Xu RK, Zhang H (2011). The forms of alkalis in the biochar produced from crop residues at different temperatures. Bioresour. Technol..

[77] Artelle LYHW, Odgers JAER (2010). Immobilization of heavy metal ions ( Cu II, Cd II, Ni II, and Pb II ) by Broiler Litter-derived biochars in water and soil. J. Agric. Food Chem..

[78] Ahmad M (2014). Biochar as a sorbent for contaminant management in soil and water: A review. Chemosphere.

[79] McGrath SP, Zhao FJ, Lombi E (2001). Plant and rhizosphere processes involved in phytoremediation of metal-contaminated soils. Plant Soil.

[80] Lasat MM (2002). Phytoextraction of toxic metals: A review of biological mechanisms. J. Environ. Qual..

[81] Dworkin M, Foster JW (1958). Experiments with some microorganisms which utilize ethane and hydrogen. J. Bacteriol..

[82] Pan Y (2016). Solubility of trace metals in two contaminated paddy soils exposed to alternating flooding and drainage. Geoderma.

[83] Awashthi, S. K. *Prevention of Food Adultration*. (Ashoka Law House, 2000).

[84] Moodie CD, Smith HW, Creery RAM (1959). Laboratory Manual for Soil Fertility.

[85] Walkley A, Black IA (1934). An examination of the degtjareff method for determining soil organic matter, and a proposed modification of the chromic acid titration method. Soil Sci..

[86] Jackson MC (1962). Soil Chemical Analysis.

[87] Watanabe FS, Olsen SR (1965). Test of an ascorbic acid method for determining phosphorus in water and NaHCO_3_ extracts from soil1. Soil Sci. Soc. Am. J..

[88] Lindsay WL, Norvell WA (1978). a DTPA soil test for zinc, iron, manganese and copper. Soil Sci. Soc. Am. J..

[89] Younis U (2015). Growth, survival, and heavy metal (Cd and Ni) uptake of spinach (*Spinacia oleracea*) and fenugreek (*Trigonella corniculata*) in a biochar-amended sewage-irrigated contaminated soil. J. Plant Nutr. Soil Sci..

[90] Chapman HD, Pratt PF (1961). Methods of Analysis for Soils, Plants and Water.

[91] Jones, J. B., WolfH, B. & Mills, H. A. *Plant Analysis Handbook: A Practical Sampling, Preparation, Analysis, and Interpretation Guide*. (Micro-Macro Publishing Inc., 1991).

[92] Nadeem F (2013). Qualitative and chemical analysis of rice kernel to time of application of phosphorus in combination with zinc under anaerobic conditions. Asian J. Agric. Biol..

[93] Steel, R. G., Torrie, J. H. & Dickey, D. A. *Principles and Procedures of Statistics: A Biometrical Approach*. (McGraw Hill Book International Co., 1997).

