# Author Correction: Potential role of compost mixed biochar with rhizobacteria in mitigating lead toxicity in spinach

**DOI:** 10.1038/s41598-020-73325-4

**Published:** 2020-10-08

**Authors:** Muhammad Zafar-ul-Hye, Muhammad Tahzeeb-ul-Hassan, Muhammad Abid, Shah Fahad, Martin Brtnicky, Tereza Dokulilova, Rahul Datta, Subhan Danish

**Affiliations:** 1 Department of Soil Science, Faculty of Agricultural Sciences and Technology, Bahauddin Zakariya University, Multan, 60800 Punjab Pakistan; 2 Department of Agronomy, The University of Haripur, Haripur, 22620 Pakistan; 3 College of Plant Sciences and Technology, Huazhong Agriculture University, Wuhan, China; 4 Department of Agrochemistry, Soil Science, Microbiology and Plant Nutrition, Faculty of AgriSciences, Mendel University in Brno, Zemedelska 1, 61300 Brno, Czech Republic; 5 Institute of Chemistry and Technology of Environmental Protection, Brno University of Technology, Faculty of Chemistry, Purkynova 118, 62100 Brno, Czech Republic

Correction to: *Scientific Reports* 10.1038/s41598-020-69183-9, published online 22 July 2020

In the original version of this Article, Shah Fahad was incorrectly affiliated with ‘Department of Agriculture, The University of Swabi, Khyber Pakhtunkhwa, Pakistan’. The correct affiliations are listed below.

Department of Agronomy, The University of Haripur, Haripur 22620, Pakistan

College of Plant Sciences and Technology, Huazhong Agriculture University, Wuhan, China

This error has now been corrected in the PDF and HTML versions of the Article.

