# Supplementary material for: Potential role of compost mixed biochar with rhizobacteria in mitigating lead toxicity in spinach
Source: Sci Rep. 2020 Jul 22;10:12159. doi: 10.1038/s41598-020-69183-9 (PMC7376197; doi:10.1038/s41598-020-69183-9)
Supplement: Supplementary file 1 — Supplementary Figures [file 41598_2020_69183_MOESM1_ESM.pdf]

Potential role of biochar mixed compost with rhizobacteria in mitigating lead toxicity in spinach

Muhammad Zafar-ul-Hye[1], Muhammad Tahzeeb-ul-Hassan[1], Muhammad Abid[1], Fahad Shah[2,3], Martin Brtnicky[4,5], Tereza Dokulilova[4], Rahul Datta[4] & Subhan Danish[1]

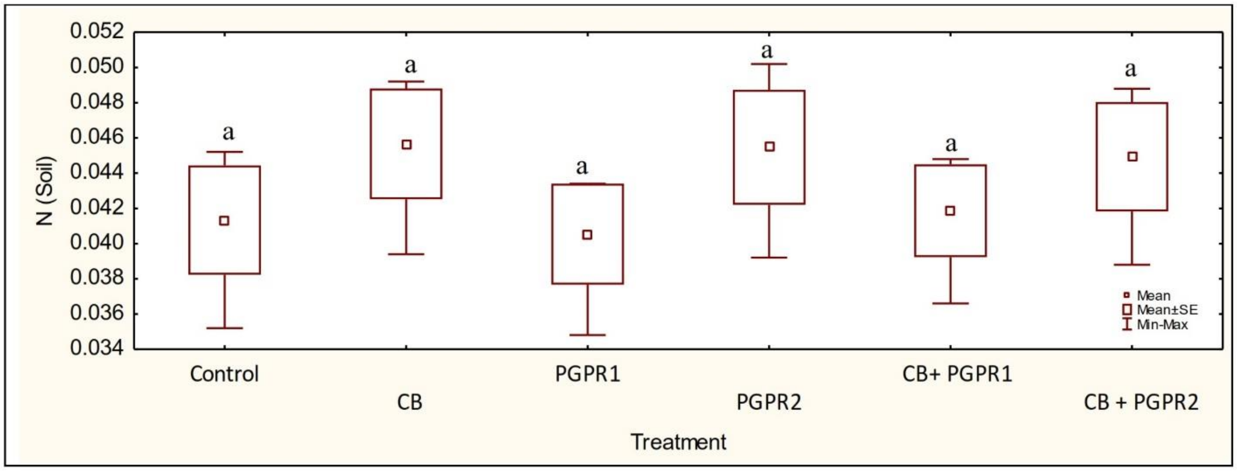

Fig. S1-Soil N (%) value of soil treated with with CB, PGPR1, PGPR2 and their combination with CB. Different small letters express significant differences.

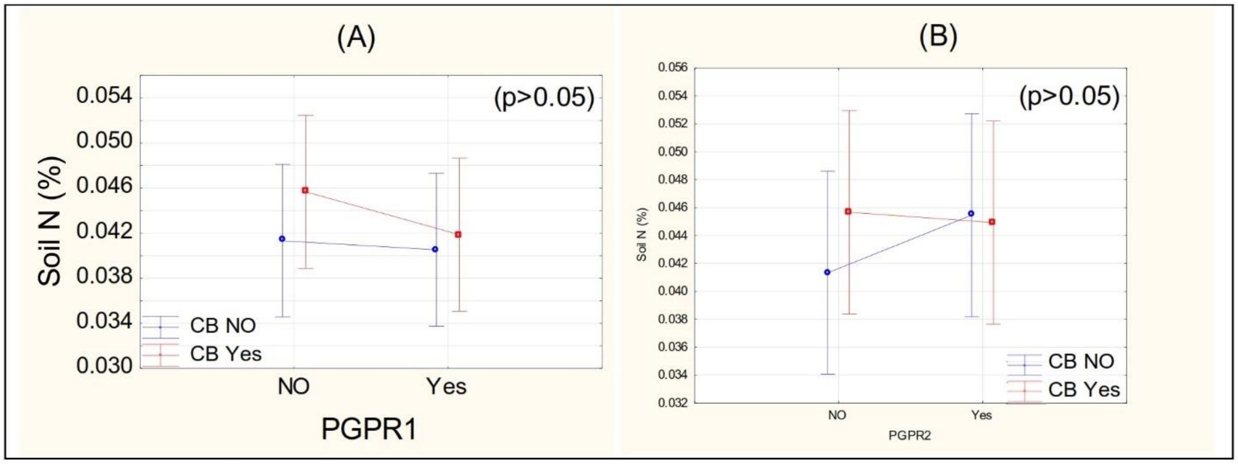

Fig. S2-Interaction graph of **(A)** PGPR1 and CB **(B)** PGPR2 and CB, for soil Soil N (%). Two factor ANOVA was conducted separately to test the interaction effect of PGPR1 and PGPR2 with CB for soil Soil N (%). PGPR1 (**Figure A**) and PGPR2 (**Figure B**) don't show any interaction with CB and also no individual main effect of either treatment were found.

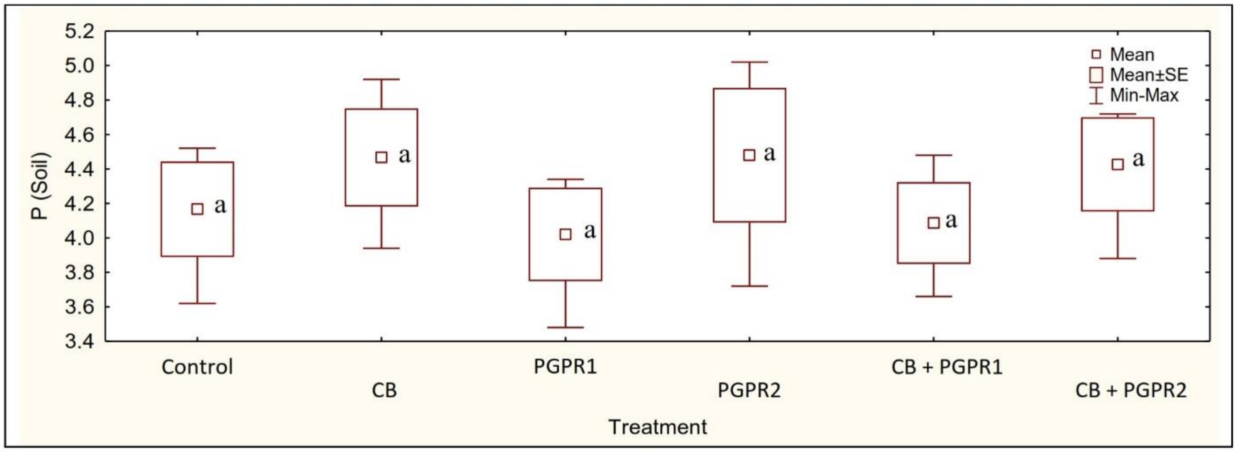

Fig. S3-P value (mg kg^{-1}) of soil treated with with CB, PGPR1, PGPR2 and their combination with CB. Different small letters express significant differences.

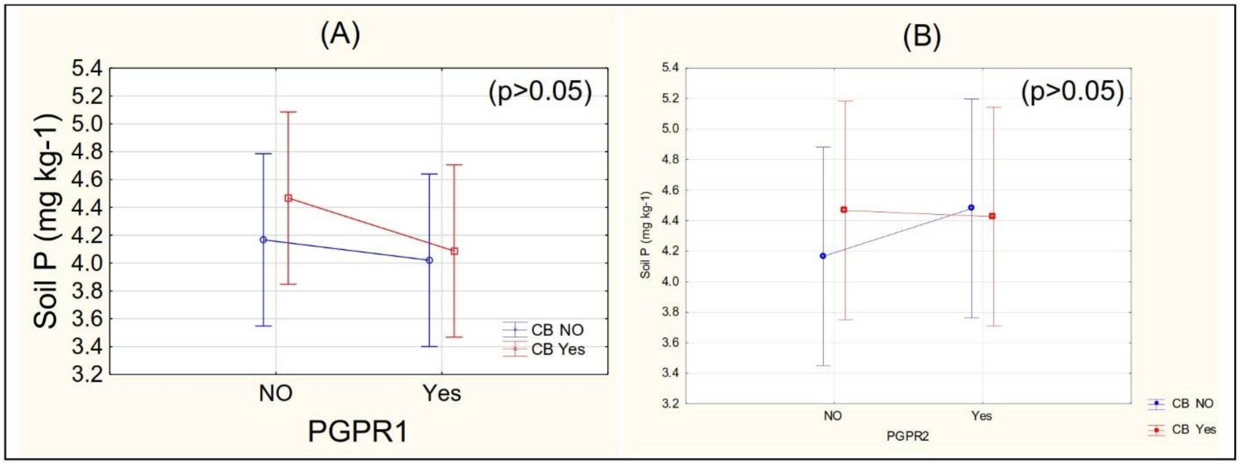

Fig. S4-Interaction graph of **(A)** PGPR1 and CB **(B)** PGPR2 and CB, for Soil P (mg kg^{-1}).

Two factor ANOVA was conducted separately to test the interaction effect of PGPR1 and PGPR2 with CB. PGPR1 (**Figure A**) and PGPR2 (**Figure B**) don't show any interaction with CB and also no individual main effect of either treatment were found.

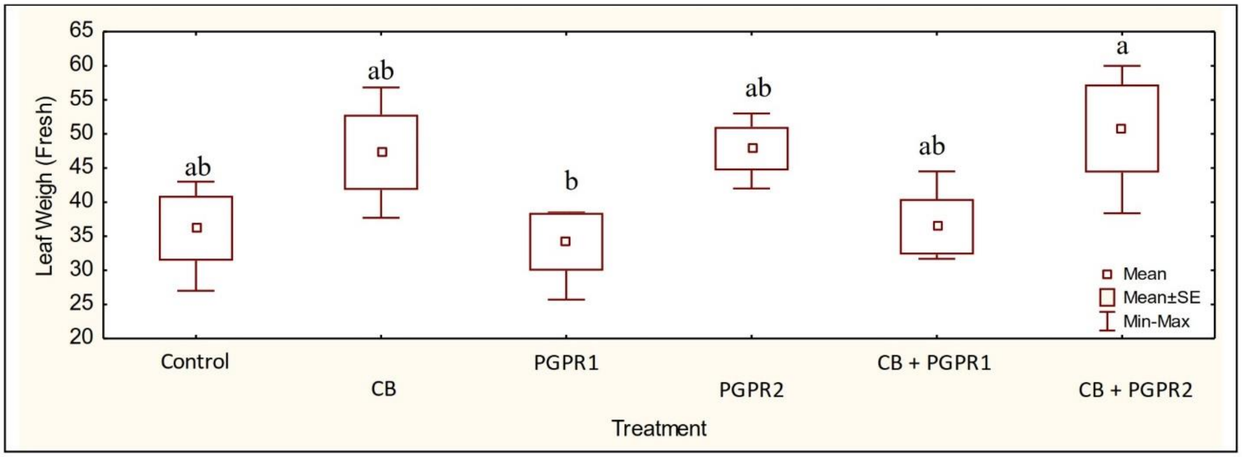

Fig. S5-Fresh leaf weight value (g) of spinach plant treated with with CB, PGPR1, PGPR2 and their combination with CB. Different small letters express significant differences.

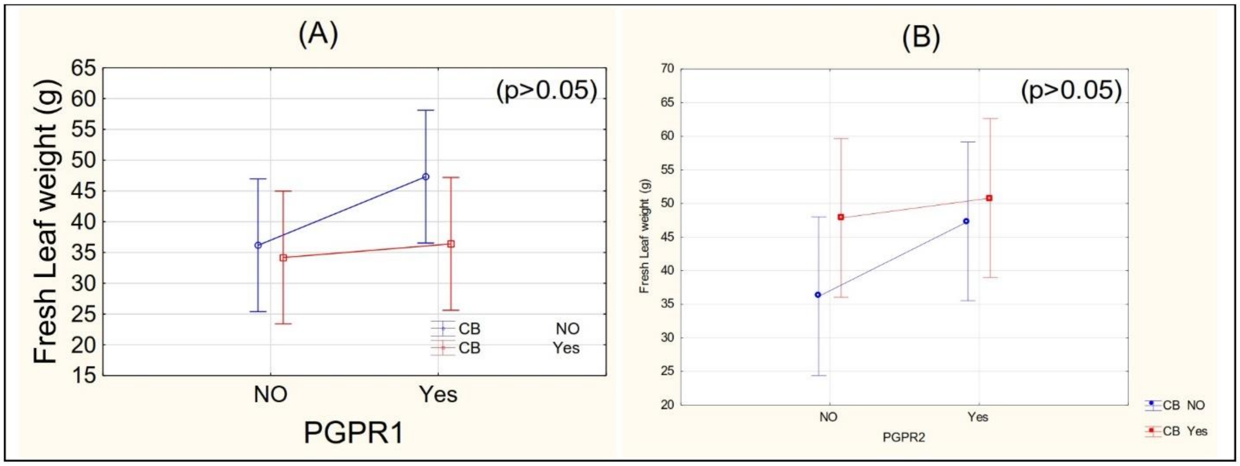

Fig. S6-Interaction graph of **(A)** PGPR1 and CB **(B)** PGPR2 and CB, for fresh leaf weight (g) of spinach plant. Two factor ANOVA was conducted separately to test the interaction effect of PGPR1 and PGPR2 with CB for fresh leaf weight (g) of spinach plant. PGPR1 (**Figure A**) and PGPR2 (**Figure B**) don't show any interaction with CB and also no individual main effect of either treatment were found.

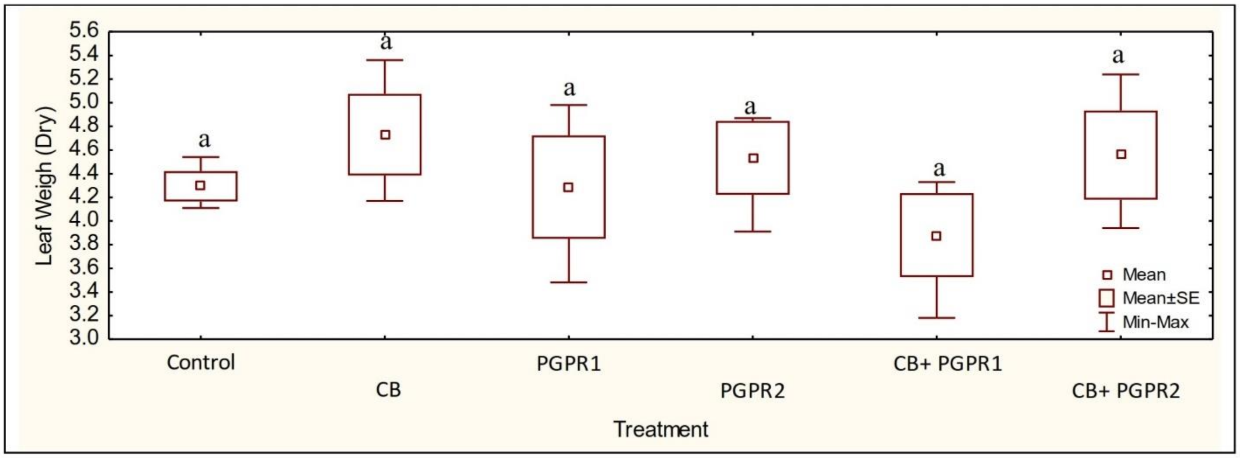

Fig. S7-Dry leaf weight (g) value of spinach plant of soil treated with with CB, PGPR1, PGPR2 and their combination with CB. Different small letters express significant differences.

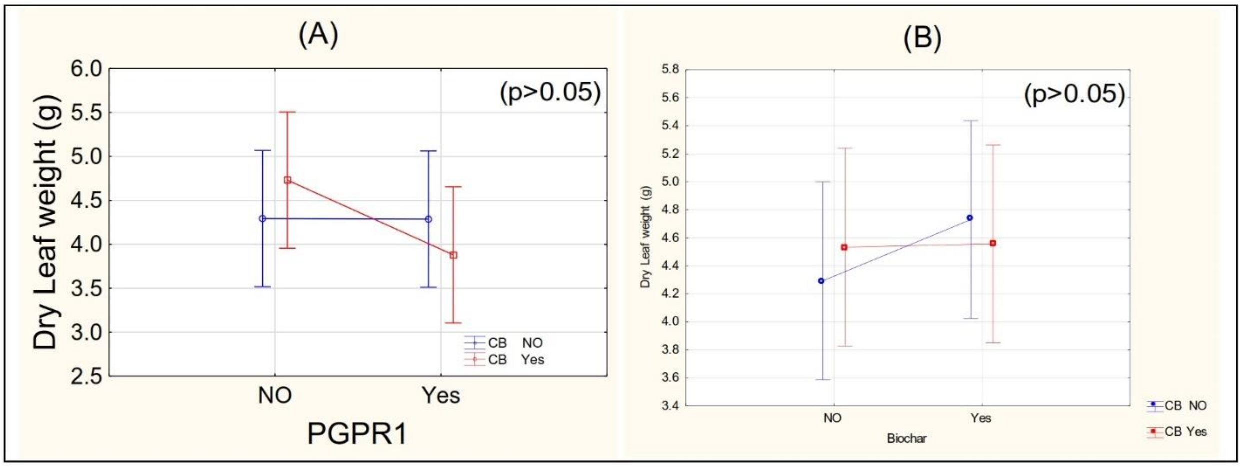

Fig. S8-Interaction graph of **(A)** PGPR1 and CB **(B)** PGPR2 and CB, for Dry leaf weight (g) value of spinach plant. Two factor ANOVA was conducted separately to test the interaction effect of PGPR1 and PGPR2 with CB on dry leaf weight (g) value of spinach plant. PGPR1 (**Figure A**) and PGPR2 (**Figure B**) don’t show any interaction with CB and also no individual main effect of either treatment were found.

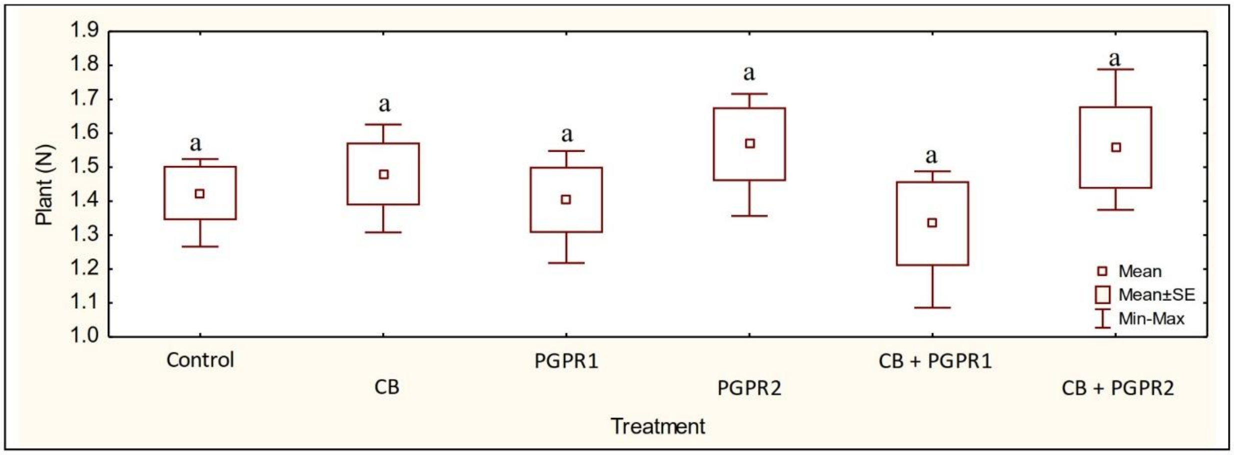

Fig. S9-Plant N value (%) of soil treated with with CB, PGPR1, PGPR2 and their combination with CB. Different small letters express significant differences.

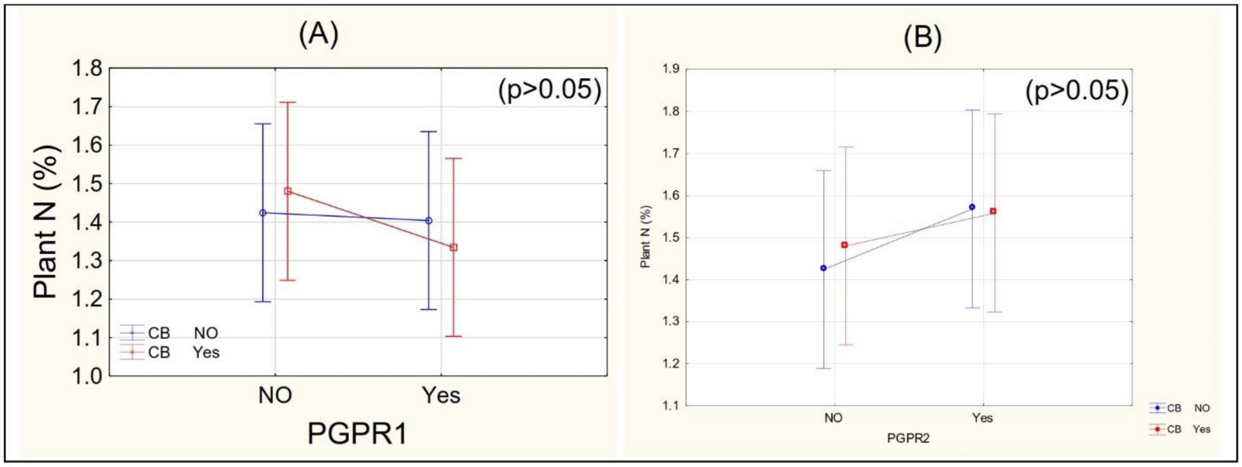

Fig. S10-Interaction graph of **(A)** PGPR1 and CB **(B)** PGPR2 and CB, for plant N (%).

Two factor ANOVA was conducted separately to test the interaction effect of PGPR1 and PGPR2 with CB on plant N (%). PGPR1 (**Figure A**) and PGPR2 (**Figure B**) don't show any interaction with CB and also no individual main effect of either treatment were found.

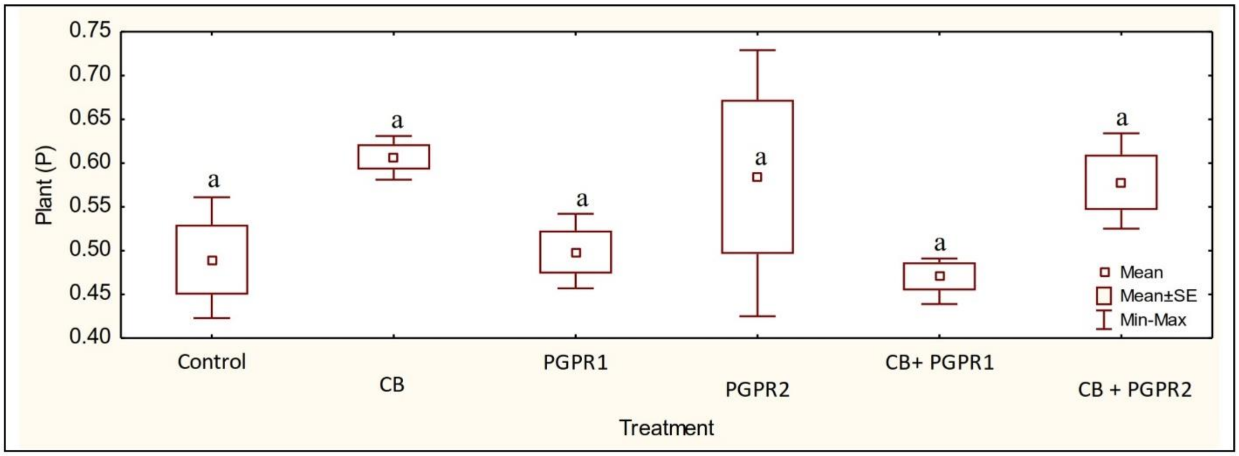

Fig. S11-Plant P (%) value of soil treated with with CB, PGPR1, PGPR2 and their combination with CB. Different small letters express significant differences.

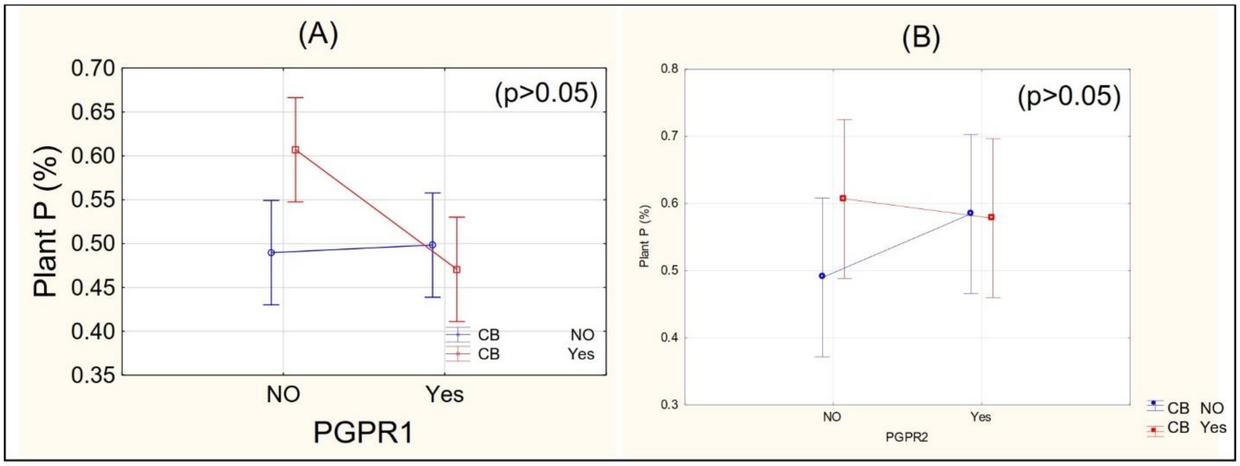

Fig. S12-Interaction graph of **(A)** PGPR1 and CB **(B)** PGPR2 and CB, for Plant P value (%).

Two factor ANOVA was conducted separately to test the interaction effect of PGPR1 and PGPR2 with CB on plant N (%). PGPR1 (**Figure A**) and PGPR2 (**Figure B**) don't show any interaction with CB and also no individual main effect of either treatment were found.

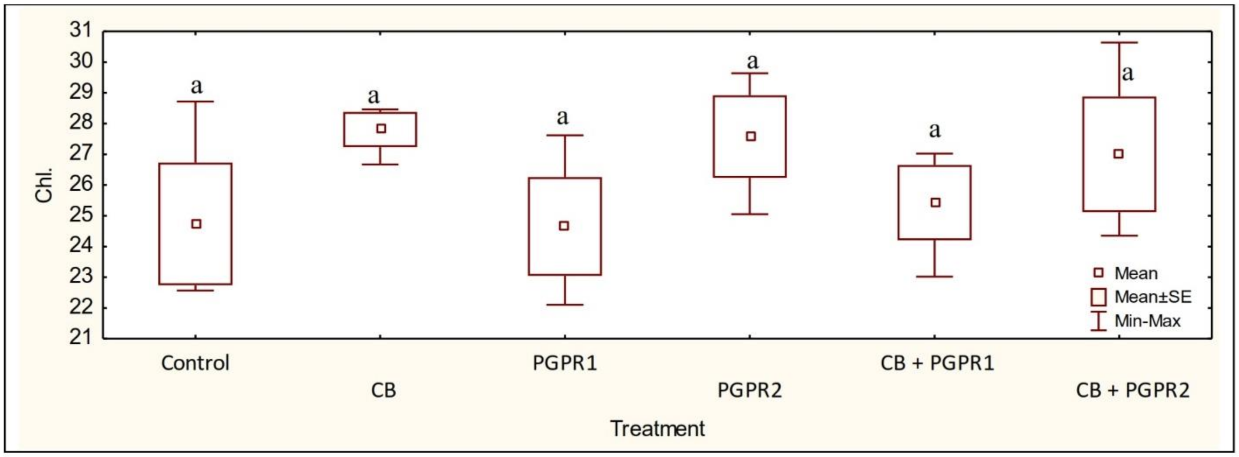

Fig. S13-Chlorophyll amount (mg g-1) of spinach plant treated with with CB, PGPR1, PGPR2 and their combination with CB. Different small letters express significant differences.

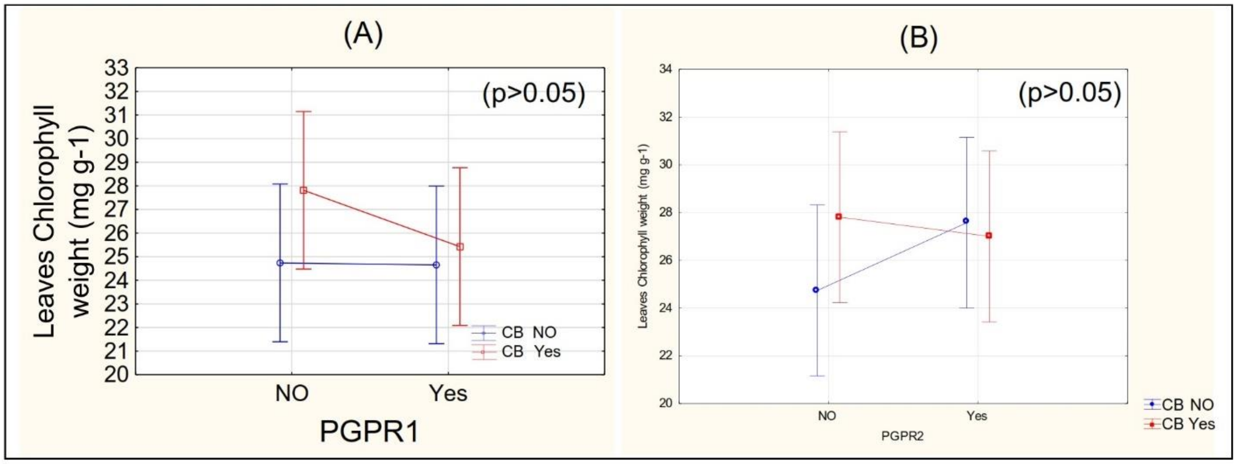

Fig. S14-Interaction graph of **(A)** PGPR1 and CB **(B)** PGPR2 and CB, for Chlorophyll amount (mg g^{-1}) of spinach plant

Two factor ANOVA was conducted separately to test the interaction effect of PGPR1 and PGPR2 with CB for Chlorophyll amount (mg g^{-1}) in spinach plant. PGPR1 (**Figure A**) and PGPR2 (**Figure B**) don't show any interaction with CB and also no individual main effect of either treatment were found.

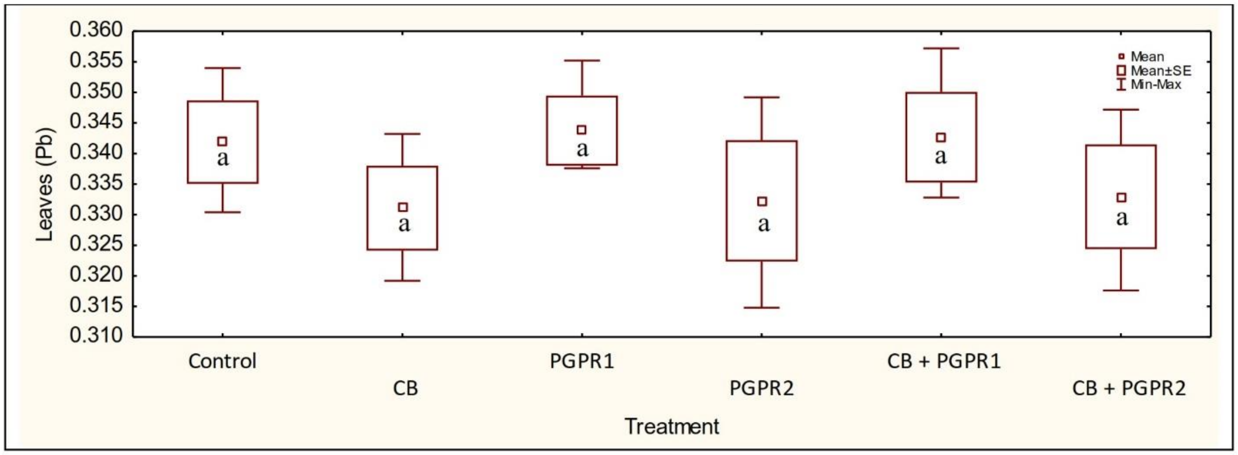

Fig. S15. Leaf Pb value (µg g^{-1}) of spinach plant treated with with CB, PGPR1, PGPR2 and their combination with CB. Different small letters express significant differences.

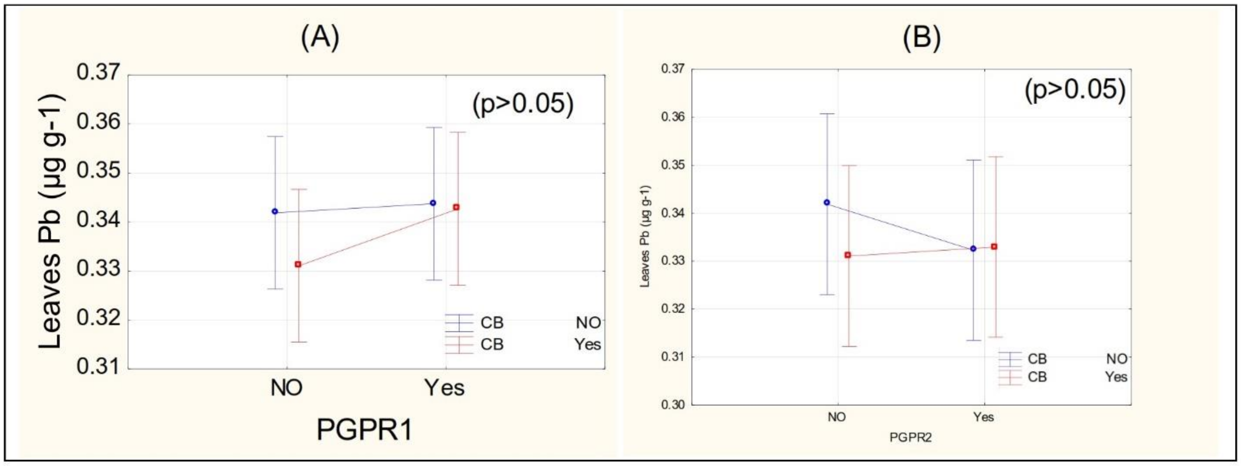

Fig. S16. Interaction graph of **(A)** PGPR1 and CB **(B)** PGPR2 and CB, for Leaf Pb value (µg g^{-1}) of spinach plant.

Two factor ANOVA was conducted separately to test the interaction effect of PGPR1 and PGPR2 with CB on Leaf Pb value. Result confirm that PGPR1 (**Figure A**) and PGPR2 (**Figure B**) don't show any interaction with CB and also no individual main effect of either treatment were found.
